# Unified Discontinuous Galerkin Analysis of a Thermo/Poro-viscoelasticity Model

**DOI:** 10.1007/s10915-025-03016-7

**Published:** 2025-09-02

**Authors:** Stefano Bonetti, Mattia Corti

**Affiliations:** MOX-Dipartimento di Matematica, Politecnico di Milano, Piazza Leonardo da Vinci 32, 20133 Milan, Italy

**Keywords:** Discontinuous Galerkin, Polytopal methods, Poro-viscoelasticity, Thermo-viscoelasticity, 65N30, 65N22, 65N12, 76S05

## Abstract

We present and analyze a discontinuous Galerkin method for the numerical modeling of a Kelvin-Voigt thermo/poro-viscoelastic problem. We present the derivation of the model and we develop a stability analysis in the continuous setting that holds both for the full inertial and quasi-static problems and that is robust with respect to most of the physical parameters of the problem. For spatial discretization, we propose an arbitrary-order weighted symmetric interior penalty scheme that supports general polytopal grids and is robust with respect to strong heterogeneities in the model coefficients. For the semi-discrete problem, we prove the extension of the stability result demonstrated in the continuous setting and we provide an a-priori error estimate. A wide set of numerical simulations is presented to assess the convergence and robustness properties of the proposed method. Moreover, we test the scheme with literature and physically sound test cases for *proof-of-concept* applications in the geophysical context.

## Introduction

In recent years, there has been an increasing interest in studying the poroelasticity equations [17, 21, 47, 55, 56, 65]. The equations of linear poroelasticity are commonly known as Biot’s equations, and they find origin in the works of Biot [13] and Terzaghi [64]. This model aims to study and describe the interaction between the fluid flow and elastic deformation within a porous medium. This problem was initially associated with geophysical applications, where the subsoil is modeled as a fully saturated poroelastic material (examples of application can be found, e.g., in [30, 36, 57]). Through the years, the classical poroelastic equations have been enhanced to couple them with other physical phenomena by including quantities that may influence (and may be influenced by) the fluid flow and the elastic deformation. Some examples can be found in poroelasto-acoustic coupling [2], which finds application in the context of earthquakes, and in the thermo-poroelasticity theory [24, 25], which is used for modeling geothermal energy production procedures and greenhouse gas sequestration.

A growing interest in the poroelasticity field has been motivated by its application to biological tissues. Indeed, organs, bones, and engineered tissue scaffolds can be modeled starting from the poroelasticity theory [15, 16, 48]. Interesting results in the biological modeling, for example, in the brain context, can be obtained using the linear poroelasticity model [31]. More sophisticated multiple-network poroelastic theory [33] considers multiple fluid compartments and, consequently, coupling terms coming from the interaction between different fluids.

However, biological tissues typically exhibit both elastic and viscoelastic behavior due to the combined action of elastin and collagen [50]. Linear viscoelastic effects can be incorporated into traditional linear Biot dynamics by considering the viscoelastic strain and possibly adjusting the formula for the local fluid content accordingly (depending on the specific scenario considered, either focusing on incompressible or compressible constituents). Some early works on poro-viscoelastic solids and their modeling are [34, 58]. This work considers the simplest (linear) visco-elasticity inclusion: the Kelvin-Voigt type [14, 18]. We remark that the viscoelastic effects may be of interest also for geophysical applications. Indeed, traditionally, they were considered in the displacement dynamics by invoking the so-called secondary consolidation [44, 51], for instance, for studies on clays.

This paper uses the full inertial Biot’s system for describing poro-viscoelasticity, which is formally equivalent to thermoelasticity and thermo-viscoelasticity problems [60]. For instance, fully dynamic thermoelasticity can be used in the context of earthquake modeling. Indeed, a mechanical source of elastic waves induces a temperature field whose heat flow equalizes the temperature difference with the surroundings, giving rise to energy dissipation. On the other hand, a heat source generates elastic or viscoelastic waves [28]. Some *proof-of-concept* numerical results in the direction of thermoelasticity are presented in this paper. Including viscous effects in the thermoelasticity theory may be useful for describing the behavior of the elastomers under large strains [23].

This paper aims to provide a general and unified framework for studying thermo/poro-viscoelasticity problems in both their quasi-static and dynamic forms (i.e., considering inertial terms). Specifically, the treatment of the model problem highlights which terms are more or less significant depending on the values of the physical parameters and, consequently, on the reference application. Second, the stability analysis of the problem is developed to have only weak constraints on the model parameters; in particular, it is designed to be robust to the presence of the inertial terms within the two equations and the parameters describing the viscoelasticity characteristics in the model problem. We notice that the stability estimate obtained in this paper is not robust with respect to the conductivity coefficient, the second Lamé parameter and the first viscoelasticity retardation time. However, in the numerical results section, we observe that the proposed scheme is (at least) numerically robust with respect to low values of these model parameters.

For the spatial discretization of the problem, we adopt a discontinuous Galerkin finite element method on polytopal grids (PolyDG) [1, 26]. The PolyDG schemes are appealing because of their geometrical flexibility, facilitating local mesh refinement and coarsening. Moreover, they allow us to efficiently handle highly heterogeneous media by better representing inner discontinuities. Geometrical flexibility is a desirable feature in both the geophysical and biological contexts, where discretization should capture peculiar features of the domain without dramatically affecting the number of elements. Another advantage of polytopal discretization techniques is the possibility of exploiting arbitrary- and, in particular, high-order approximations. In second-order hyperbolic problems, high-order discretizations minimize the dispersion and diffusive phenomena. However, we remark that for subsurface applications, we may not have the regularity needed to justify high-order discretizations due to the large variabilities of the model’s coefficients [41, 62]. The proposed method’s arbitrary-order accuracy, combined with its geometric flexibility, allows using *hp*-refinement techniques, taking full advantage of agglomeration techniques and reducing the overall computational cost.

Examples of PolyDG schemes can be found in [2] for poroelasticity, in [4, 19, 20] for thermo-hydro-mechanics (both in the quasi-static and fully-dynamic regimes), and in [33] for multiple-network poroelasticity. Moreover, in [2, 3, 35], PolyDG methods for wave propagation problems in porous media are analyzed. Last, in [10], a discontinuous Galerkin method for the Kelvin-Voigt viscoelastic fluid model is proposed. In the literature, other discretization strategies for the poroelasticity problems include, e.g., finite Volume methods [12, 59], Hybrid Finite Volume method [8], Hybrid High-Order [22], Hybridizable Discontinuous Galerkin [42], lowest order Raviart-Thomas coupled with conforming finite element methods [21], and eXtended finite element method [46].

The major highlights of this paper are: *(a)* a detailed discussion of the model problem, in which the importance of the inertial terms as a function of the physical parameters of the problem is highlighted; *(b)* an analysis covering both the static problem case and the fully inertial problem that investigates the stability of the problem under mild assumptions on the problem coefficients (for both the continuous and the semi-discrete problem) and an a-priori error estimate (for the semi-discrete problem); and *(c)* a wide set of numerical simulations that are intended to prove the convergence and robustness properties of the method, test the method against literature benchmarks, and explore the applicability of the method in *physically-sound* test cases.

The rest of the paper is organized as follows: the model problem, its derivation, and its weak formulation are reported in Section 2. In Section 3, we prove the stability of the continuous problem. In Section 4, we design the discretization of the problem. In particular, in Section 4.2, we detail the PolyDG space discretization, and in Section 4.4, we show the time-discretization techniques we exploit for the hyperbolic-hyperbolic case and the hyperbolic-parabolic case. Moreover, we extend the stability result obtained in Section 3 for the discrete setting and we derive an a-priori error estimate. Last, we report numerical results in Section 5. Namely, we start by assessing the method’s performance in terms of convergence properties and robustness, and then we address benchmark and physically sound test cases.

## Model Problem

Let $\Omega \subset {\mathbb {R}}^d$, $d = \{2;3\}$, be an open, polygonal/polyhedral domain with Lipschitz boundary $\partial \Omega$. Given a final time $T_f > 0$, the problem reads: *find *$({\textbf{u}}, \varphi )$* such that:*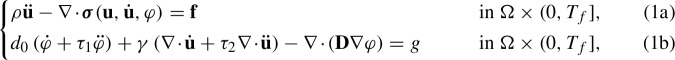 where the unknowns $({\textbf{u}},\varphi )$ stand for the solid displacement and a generalized pressure variable. In problem (1), equation (1a) represents the momentum conservation law. At the same time, equation (1b) can represent both a mass conservation or an energy conservation law based on the physical interpretation given to the variable $\varphi$, which we discuss later in this section. Thus, the terms ${\textbf{f}}$ and *g* are source terms that represent a body force and a mass or energy source, respectively. Finally, problem (1) is supplemented by imposing suitable boundary and initial conditions.

In (1) and in the rest of the article, we use the short-hand notation ${\dot{\psi }}$ and $\ddot{\psi }$ for denoting the first and second partial derivatives with respect to time of a function $\psi :\Omega \times (0,T_f]\rightarrow {\mathbb {R}}$, respectively.

The constitutive law for the total stress tensor $\varvec{\sigma }$ is obtained as in [34] under the small deformations assumption, taking into account the isotropic effect of the generalized-pressure field on the porous matrix: where ${\textbf{I}}$ is the identity tensor and $\varvec{\epsilon }({\textbf{u}})= \frac{1}{2}(\nabla {\textbf{u}} + \nabla {\textbf{u}}^T)$ is the strain tensor. Moreover, the dependency on the velocity of the deformation ${\textbf{u}}$ models the secondary consolidation phenomena in poroelasticity for coil applications [11].

The generalized pressure variable $\varphi$ can be considered a pressure in a poro-(visco)elasticity framework or a temperature in a thermo-(visco)elasticity one. In the first case, the second equation rises from the linearized equation of mass conservation [34]:

$$\begin{aligned} d_0{\dot{\varphi }}+\gamma (\nabla \cdot \dot{{\textbf{u}}}) + \nabla \cdot {\textbf{w}} = {\tilde{g}}, \end{aligned}$$

where ${\textbf{w}}$ is the filtration velocity, $d_0$ is the specific storage coefficient, and $\gamma$ is the Biot-Willis parameter [34]. Then, equation (1b) is obtained by adopting a Darcy law that considers the acceleration of the fluid [52]:

2 $$\begin{aligned} \dfrac{\rho _f}{\phi } {\textbf{K}}\dot{{\textbf{w}}}=- {\textbf{K}}\nabla \varphi - {\textbf{w}}, \end{aligned}$$

where $\rho _f$ is the fluid density, $\phi$ is the porosity (the ratio between the void space in a porous medium and its whole volume such that $0< \phi _0 \le \phi \le \phi _1 < 1$ [61]), ${\textbf{K}}$ is the permeability divided by the dynamic fluid viscosity and ${\tilde{g}}$ is the forcing term. In particular, to obtain the formulation in equation (1b), we need to define $\tau _1 = \rho _f {\textbf{K}} \phi ^{-1}$, $\tau _2 = \rho _f {\textbf{K}} \phi ^{-1}$, ${\textbf{D}}={\textbf{K}}$, $g=\tau _1\dot{{\tilde{g}}}+{\tilde{g}}$. In the dynamical Darcy law in Equation (2), we neglect the convection term ($({\textbf{w}}\cdot \nabla ){\textbf{w}}$), because it is generally small and negligible in most applications [52].

On the contrary, in the thermoelastic case, considering $\varphi$ as a temperature, equation (1b) can be derived from the linearized equation of energy conservation [34]:

$$\begin{aligned} d_0{\dot{\varphi }}+\gamma (\nabla \cdot \dot{{\textbf{u}}}) + \nabla \cdot {\textbf{q}} = {\tilde{g}}, \end{aligned}$$

where ${\textbf{q}}$ is the heat flux, $d_0$ is the thermal dilatation coefficient, and $\gamma$ is the thermal stress parameter [34]. Then, equation (1b) is obtained by adopting a Maxwell-Vernotte-Cattaneo law that considers a relaxation time in the heat conduction equation [52]:

$$\begin{aligned} \tau \dot{{\textbf{q}}}= -\varvec{\Theta } \nabla \varphi - {\textbf{q}}, \end{aligned}$$

where $\tau$ is the relaxation time, $\varvec{\Theta }$ is the effective thermal conductivity and ${\tilde{g}}$ is the forcing term. In particular, to obtain the formulation in equation (1b), we need to define $\tau _1 = \tau$, $\tau _2 = \tau$, ${\textbf{D}}=\varvec{\Theta }$, $g=\tau _1\dot{{\tilde{g}}}+{\tilde{g}}$. In both cases, the choice of considering two different values $\tau _1$ and $\tau _2$ in the final model is for generalization purposes.

The coefficients appearing in (1), along with their unit of measure and physical meaning in poroelasticity or thermoelasticity frameworks, are reported in Table 1:

**Table 1:** Model coefficients appearing in problem (1) with explicit indication of the associated framework of the description: poroelasticity (P) or thermoelasticity (T). Where no indication is provided, the unit and the description are valid for both frameworks

**Parameter**	**Unit**	**Quantity**
$\rho _f$	$\hbox {kg/m}^{3}$	Saturating fluid density
$\rho _s$	$\hbox {kg/m}^{3}$	Solid matrix density
$\phi$	-	**P:** Porosity
$\rho$	$\hbox {kgm}^{-3}$	**P:** Density $\rho = \phi \rho _f + (1 - \phi ) \rho _s$
	$\hbox {kgm}^{-3}$	**T:** Density $\rho = \rho _s$
$\mu , \lambda$	Pa	Lamé parameters
$\delta _1, \delta _2$	s	Viscoelasticity retardation times
$\gamma$	-	**P:** Biot–Willis constant
	PaK$^{-1}$	**T:** Thermal stress coefficient
$d_0$	Pa$^{-1}$	**P:** Specific storage coefficient
	$\hbox {PaK}^{-2}$	**T:** Thermal capacity
${\textbf{D}}$	m^2^Pa$^{-1}$s$^{-1}$	**P:** Permeability divided by dynamic fluid viscosity
	m^2^K$^{-1}$^2^s$^{-1}$	**T:** Effective thermal conductivity
$\tau _1$,$\tau _2$	s	**P:** Relaxation times
	s	**T:** Maxwell-Vernotte-Cattaneo relaxation times

As already mentioned, from the general formulation of equation (1) we can recover some specific models.

### Poroelasticity model

The classical poroelastic Biot problem [13], can be recovered by taking the pressure field $p=\varphi$ and considering $\tau _1=\tau _2=\delta _1=\delta _2=0$.

$$\begin{aligned} \left\{ \begin{aligned}&\rho \ddot{{\textbf{u}}} - 2\nabla \hspace{-1.38885pt}\cdot \hspace{-1.38885pt}{(\mu \varvec{\epsilon }({\textbf{u}}))} - \nabla {(\lambda \nabla \cdot {\textbf{u}})} + \nabla {(\gamma p)} = {\textbf{f}} \qquad  &   \text {in }\Omega \times (0,T_f], \\&d_0 {\dot{p}}+ \gamma \left( \nabla \hspace{-1.38885pt}\cdot \hspace{-1.38885pt}{\dot{{\textbf{u}}}} \right) - \nabla \hspace{-1.38885pt}\cdot \hspace{-1.38885pt}{\left( {\textbf{D}} \nabla p \right) } = g \qquad  &   \text {in }\Omega \times (0,T_f]. \end{aligned} \right. \end{aligned}$$

#### Thermoelasticity model

The thermoelasticity problem with the Maxwell-Vernotte-Cattaneo relaxation law can be recovered by taking the temperature field $T=\varphi$ and considering $\tau _1=\tau _2=\tau$ and $\delta _1=\delta _2=0$.

$$\begin{aligned} \left\{ \begin{aligned}&\rho \ddot{{\textbf{u}}} - 2\nabla \hspace{-1.38885pt}\cdot \hspace{-1.38885pt}{(\mu \varvec{\epsilon }({\textbf{u}}))} - \nabla {(\lambda \nabla \cdot {\textbf{u}})} + \nabla {(\gamma T)} = {\textbf{f}} \qquad  &   \text {in }\Omega \times (0,T_f], \\&d_0 \left( {\dot{T}} + \tau {\ddot{T}} \right) + \gamma \left( \nabla \hspace{-1.38885pt}\cdot \hspace{-1.38885pt}{\dot{{\textbf{u}}}} + \tau \nabla \hspace{-1.38885pt}\cdot \hspace{-1.38885pt}{\ddot{{\textbf{u}}}} \right) - \nabla \hspace{-1.38885pt}\cdot \hspace{-1.38885pt}{\left( {\textbf{D}} \nabla T \right) } = g \qquad  &   \text {in }\Omega \times (0,T_f]. \end{aligned} \right. \end{aligned}$$

By taking also $\tau =0$, we recover the formulation with the Fourier law for the temperature.

#### Poro-viscoelasticity model

The classical poro-viscoelasticity problem [18] can be recovered by taking the pressure field $p=\varphi$ and considering $\tau _1=\tau _2=0$.

$$\begin{aligned} \left\{ \begin{aligned}&\rho \ddot{{\textbf{u}}} - \nabla \hspace{-1.38885pt}\cdot \hspace{-1.38885pt}{( 2 \mu (\varvec{\epsilon }({\textbf{u}}) + \delta _1 \varvec{\epsilon }(\dot{{\textbf{u}}})))} - \nabla {\left( \lambda (\nabla \cdot {\textbf{u}} + \delta _2 \nabla \cdot \dot{{\textbf{u}}}\right) )} + \nabla {(\gamma p)} = {\textbf{f}} \quad  &   \text {in }\Omega \times (0,T_f], \\&d_0 {\dot{p}} + \gamma \nabla \hspace{-1.38885pt}\cdot \hspace{-1.38885pt}{\dot{{\textbf{u}}}} - \nabla \hspace{-1.38885pt}\cdot \hspace{-1.38885pt}{\left( {\textbf{D}} \nabla p \right) } = g \quad  &   \text {in }\Omega \times (0,T_f]. \end{aligned} \right. \end{aligned}$$

By taking $\delta _1=0$, we recover the secondary consolidation poroelastic model.

### Weak Formulation

Before presenting the variational formulation of problem (1), we introduce the required notation. For $X\subseteq \Omega$, we denote by $L^p(X)$ the standard Lebesgue space of index $p\in [1, \infty ]$ and by $H^q(X)$ the Sobolev space of index $q \ge 0$ of real-valued functions defined on *X*. The notation ${\textbf{L}}^p(X)$ and ${\textbf{H}}^q(X)$ is adopted in place of $\left[ L^p(X) \right] ^d$ and $\left[ H^q(X) \right] ^d$, respectively. These spaces are equipped with natural inner products and norms denoted by $(\cdot , \cdot )_X = (\cdot , \cdot )_{L^2(X)}$ and $||\cdot ||_X = ||\cdot ||_{L^2(X)}$, with the convention that the subscript can be omitted in the case $X=\Omega$.

We denote by $\langle \cdot ,\cdot \rangle$ the duality pairing between the space $Y^*$ and *Y*; the former being the dual space of the latter. Moreover, we denote by $\Vert \cdot \Vert _*$ the dual norm in the space $Y^*$.

For the sake of brevity, in what follows, we make use of the symbol $x \lesssim y$ to denote $x \le C y$, where *C* is a positive constant independent of the discretization parameters but possibly dependent on physical coefficients and final time $T_f$.

To derive the weak formulation of problem (1), we start by providing the definition of the functional spaces that take into account the essential boundary conditions, namely

$$\begin{aligned} \begin{aligned} V&= H_0^1(\Omega ) = \left\{ \varphi \in H^1(\Omega ) \ \text {s.t.} \ \varphi _{\vert \partial \Omega } = 0 \right\} , \quad {\textbf{V}} = \left[ V \right] ^d. \end{aligned} \end{aligned}$$

Next, we multiply (1) times suitable test functions, integrate in space, and we get:

*For any time *$t \in (0, T_f]$*, find *$({\textbf{u}}, \varphi )(t) \in {\textbf{V}} \times V$* such that:*

3 $$\begin{aligned} \begin{aligned} {\mathscr {M}}_u(\ddot{{\textbf{u}}}, {\textbf{v}})&+ (2\mu \delta _1 \, \varvec{\epsilon }(\dot{{\textbf{u}}}),\varvec{\epsilon }({\textbf{v}})) + (\lambda \delta _2 \, \nabla \hspace{-1.38885pt}\cdot \hspace{-1.38885pt}{\dot{{\textbf{u}}}},\nabla \hspace{-1.38885pt}\cdot \hspace{-1.38885pt}{{\textbf{v}}}) \\&+ (2\mu \varvec{\epsilon }({\textbf{u}}),\varvec{\epsilon }({\textbf{v}})) + (\lambda \nabla \hspace{-1.38885pt}\cdot \hspace{-1.38885pt}{{\textbf{u}}},\nabla \hspace{-1.38885pt}\cdot \hspace{-1.38885pt}{{\textbf{v}}}) - (\gamma \nabla \hspace{-1.38885pt}\cdot \hspace{-1.38885pt}{{\textbf{v}}}, \varphi ) = ({\textbf{f}}, {\textbf{v}})  &   \forall {\textbf{v}}\in {\textbf{V}}, \\ {\mathscr {M}}_{\varphi , \tau _1}(\ddot{\varphi },\psi )&+ (\gamma \tau _2 \, \nabla \hspace{-1.38885pt}\cdot \hspace{-1.38885pt}{\ddot{{\textbf{u}}}}, \psi ) + {\mathscr {M}}_{\varphi }({\dot{\varphi }},\psi ) + (\gamma \nabla \hspace{-1.38885pt}\cdot \hspace{-1.38885pt}{\dot{{\textbf{u}}}}, \psi ) \\&+ ({\textbf{D}} \nabla \varphi , \nabla \psi ) = (g, \psi )  &   \forall \psi \in V, \end{aligned} \end{aligned}$$

where for every $({\textbf{u}}, \varphi ), \, ({\textbf{v}},\psi ) \in {\textbf{V}} \times V$ we have set: ${\mathscr {M}}_u({\textbf{u}}, {\textbf{v}}) = (\rho \, {\textbf{u}}, {\textbf{v}})$, ${\mathscr {M}}_{\varphi , \tau _1}(\varphi , \psi ) = (d_0 \tau _1 \, \varphi , \psi )$, and ${\mathscr {M}}_{\varphi }(\varphi , \psi ) = (d_0 \, \varphi , \psi )$.

## Stability Analysis

This section is devoted to proving the stability bounds of the continuous weak solution of problem (3). Before stating the theorem, we introduce the following auxiliary norms of the displacement ${\textbf{u}}$ in ${\textbf{V}}$:

$$\begin{aligned} \Vert {\textbf{u}}\Vert _{\textbf{V}}^2 =&\,2\Vert \sqrt{\mu }\epsilon ({\textbf{u}})\Vert ^2+\Vert \sqrt{\lambda }\nabla \cdot {\textbf{u}}\Vert ^2, \\ \Vert {\textbf{u}}\Vert _{{\textbf{V}}\delta }^2 =&\,2\Vert \sqrt{\mu \delta _1}\epsilon ({\textbf{u}})\Vert ^2+\Vert \sqrt{\lambda \delta _2}\nabla \cdot {\textbf{u}}\Vert ^2. \end{aligned}$$

Moreover, we introduce an assumption on the regularity of model parameters:

### Assumption 1

(Data regularity) We assume the following regularities for the coefficients, the forcing terms, and the initial conditions:

(*i*): $\mu ,\, \lambda ,\, \rho ,\, d_0,\, \gamma ,\, \tau _1,\, \tau _2,\, \delta _1,\, \delta _2 \in L^\infty (\Omega )$, moreover we require $\mu >0$;
(*ii*): $$\varvec{\textrm{D}}\in {\textbf{L}}^\infty (\Omega )$$ and $$\exists d>0\;\forall \varvec{\xi }\in {\mathbb {R}}^d:\; d|\varvec{\xi }|^2 \le \varvec{\xi }^\top {\textbf{D}}\varvec{\xi } \quad \forall \varvec{\xi }\in {\mathbb {R}}^d$$;
(*iii*): $${\textbf{f}}\in C^0(0,T_f; {\textbf{H}}^{-1}(\Omega ))$$;
(*iv*): $$g\in C^0(0,T_f; H^{-1}(\Omega ))$$;
(*v*): $${\textbf{u}}_0 \in {\textbf{H}}^1_0(\Omega )$$, $$\dot{{\textbf{u}}}_0 \in {\textbf{L}}^2(\Omega )$$, $$\varphi _0 \in L^2(\Omega )$$, and $${\dot{\varphi }}_0 \in L^2(\Omega )$$.

### Theorem 1

Let us consider – for any time $t\in (0,T_f]$ – $({\textbf{u}},\varphi )(t)\in {\textbf{V}}\times V$ to be the solution of the weak problem (3) with homogeneous Dirichlet boundary conditions. Under Assumption 1, then the following stability estimate holds:

$$\begin{aligned}&\Vert \sqrt{\tau _2\rho }{\textbf{u}}\Vert ^2 + \int _0^t \left( \Vert \tau _2\sqrt{\rho }\dot{{\textbf{u}}}\Vert ^2 + \Vert \sqrt{\rho }{\textbf{u}}\Vert ^2 + \left\| \dfrac{\tau _2}{2} {\textbf{u}}\right\| ^2_{\textbf{V}} + \Vert \sqrt{\tau _2}{\textbf{u}}\Vert ^2_{{\textbf{V}}\delta } + \Vert \sqrt{\tau _2 \tau _1 d_0} \varphi \Vert ^2 \right) \lesssim \\  &\Vert \sqrt{\tau _2\rho }{\textbf{u}}_0\Vert ^2 + t I_0 + \int _0^t \left( \Vert {\textbf{F}}\Vert ^2_{*} + \dfrac{\left\| \sqrt{\tau _2} G\right\| ^2_{*}}{{\Vert \sqrt{{\textbf{D}}}\Vert ^2}} + t \left\| \dfrac{\tau _2{\textbf{f}}}{\sqrt{\mu \delta _1}}\right\| ^2_{*} + t\dfrac{\Vert \tau _2 g\Vert ^2_{*}}{\Vert \sqrt{{\textbf{D}}}\Vert ^2} + t\left\| \dfrac{{\textbf{F}}}{\sqrt{\mu \delta _1}}\right\| ^2_{*} + t\dfrac{\Vert G\Vert ^2_{*}}{\Vert \sqrt{{\textbf{D}}}\Vert ^2}\right) , \end{aligned}$$

where:

4 $$\begin{aligned} \begin{aligned} {\textbf{F}} =&\,\int _0^t {\textbf{f}} + \rho \dot{{\textbf{u}}}_0 - 2 \nabla \cdot \mu \delta _1\varvec{\epsilon }({\textbf{u}}_0) - \nabla (\lambda \delta _2\nabla \cdot {\textbf{u}}_0), \\ G =&\, \int _0^t g + d_0 (\varphi _0 + \tau _1 {\dot{\varphi }}_0) + \gamma (\nabla \cdot {\textbf{u}}_0+\tau _2\nabla \cdot \dot{{\textbf{u}}}_0), \\ I_0 =&\, \Vert \tau _2\sqrt{\rho }\dot{{\textbf{u}}}_0\Vert ^2 + \Vert \sqrt{\rho }{\textbf{u}}_0\Vert ^2 + \Vert \tau _2 {\textbf{u}}_0\Vert ^2_{\textbf{V}} + \Vert \sqrt{\tau _2 \tau _1 d_0} \varphi _0\Vert ^2. \end{aligned} \end{aligned}$$

In this theorem, the (hidden) stability constant is independent of the physical parameters.

### Proof

To start the proof of stability, we introduce two auxiliary variables $\Psi (t) = \int _0^t \varphi (s)\textrm{d} s$ and ${\textbf{W}}(t) = \int _0^t {\textbf{u}}(s)\textrm{d} s$. Then, we define two additional problems. The first is obtained by integrating Equation (1b) in time:

5 $$\begin{aligned} {\left\{ \begin{array}{ll} \rho \ddot{{\textbf{u}}} - 2 \nabla \hspace{-1.38885pt}\cdot \hspace{-1.38885pt}{\mu \varvec{\epsilon }({\textbf{u}})} - 2 \nabla \hspace{-1.38885pt}\cdot \hspace{-1.38885pt}{\mu \delta _1 \varvec{\epsilon }(\dot{{\textbf{u}}})} - \nabla (\lambda \nabla \cdot {\textbf{u}}) - \nabla (\lambda \delta _2 \nabla \cdot \dot{{\textbf{u}}}) + \nabla (\gamma \varphi ) = {\textbf{f}}, \\ d_0 \left( {\varphi } + \tau _1 {\dot{\varphi }} \right) + \gamma \left( \nabla \hspace{-1.38885pt}\cdot \hspace{-1.38885pt}{{{\textbf{u}}}} + \tau _2 \nabla \hspace{-1.38885pt}\cdot \hspace{-1.38885pt}{\dot{{\textbf{u}}}} \right) - \nabla \hspace{-1.38885pt}\cdot \hspace{-1.38885pt}{\left( {\textbf{D}} \nabla \Psi \right) } = G, \end{array}\right. } \end{aligned}$$

The second formulation is obtained by integrating both Equations (1a) and (1b) in time:

6 $$\begin{aligned} {\left\{ \begin{array}{ll} \rho \dot{{\textbf{u}}} - 2 \nabla \hspace{-1.38885pt}\cdot \hspace{-1.38885pt}{\mu \varvec{\epsilon }({\textbf{W}})} - 2 \nabla \hspace{-1.38885pt}\cdot \hspace{-1.38885pt}{\mu \delta _1 \varvec{\epsilon }({\textbf{u}})} - \nabla (\lambda \nabla \cdot {\textbf{W}}) - \nabla (\lambda \delta _2 \nabla \cdot {\textbf{u}}) + \nabla (\gamma \Psi ) = {\textbf{F}}, \\ d_0 \left( {\varphi } + \tau _1 {\dot{\varphi }} \right) + \gamma \left( \nabla \hspace{-1.38885pt}\cdot \hspace{-1.38885pt}{{{\textbf{u}}}} + \tau _2 \nabla \hspace{-1.38885pt}\cdot \hspace{-1.38885pt}{\dot{{\textbf{u}}}} \right) - \nabla \hspace{-1.38885pt}\cdot \hspace{-1.38885pt}{\left( {\textbf{D}} \nabla \Psi \right) } = G. \end{array}\right. } \end{aligned}$$

The two additional forcing terms ${\textbf{F}}$ and *G* are defined in Equation (4). The first step of the proof consists in obtaining the weak formulations of (5), (6). To do so, we multiply the first equations of (5), (6) times ${\textbf{v}} \in {\textbf{V}}$, and the second equations of (5), (6) times $\psi \in V$. Then, we integrate by parts and sum the contributions of the two systems of equations, respectively. The total weak formulation of (5) reads:

7 $$\begin{aligned} \begin{aligned}&(\rho \ddot{{\textbf{u}}}, {\textbf{v}}) + (d_0 \varphi , \psi ) + (d_0 \tau _1 {\dot{\varphi }}, \psi ) + (2\mu \varvec{\epsilon }({\textbf{u}}),\varvec{\epsilon }({\textbf{v}})) + (2\mu \delta _1 \varvec{\epsilon }(\dot{{\textbf{u}}}),\varvec{\epsilon }({\textbf{v}})) + (\lambda \nabla \hspace{-1.38885pt}\cdot \hspace{-1.38885pt}{{\textbf{u}}}, \nabla \hspace{-1.38885pt}\cdot \hspace{-1.38885pt}{{\textbf{v}}}) \\&\quad + (\lambda \delta _2 \nabla \hspace{-1.38885pt}\cdot \hspace{-1.38885pt}{\dot{{\textbf{u}}}}, \nabla \hspace{-1.38885pt}\cdot \hspace{-1.38885pt}{{\textbf{v}}}) + ({\textbf{D}} \nabla \Psi , \nabla \psi ) - (\varphi , \gamma \nabla \hspace{-1.38885pt}\cdot \hspace{-1.38885pt}{{\textbf{v}}}) + (\gamma \nabla \hspace{-1.38885pt}\cdot \hspace{-1.38885pt}{{{\textbf{u}}}}, \psi ) + (\gamma \tau _2 \nabla \hspace{-1.38885pt}\cdot \hspace{-1.38885pt}{\dot{{\textbf{u}}}}, \psi )\\&\quad = ({\textbf{f}}, {\textbf{v}}) + (G, \psi ) \end{aligned} \end{aligned}$$

and the total weak formulation of (6) reads: total weak formulation of (5) reads:

8 $$\begin{aligned} \begin{aligned}&(\rho \dot{{\textbf{u}}}, {\textbf{v}}) + (d_0 \varphi , \psi ) + (d_0 \tau _1 {\dot{\varphi }}, \psi ) + (2\mu \varvec{\epsilon }({\textbf{W}}),\varvec{\epsilon }({\textbf{v}})) + (2\mu \delta _1 \varvec{\epsilon }({\textbf{u}}),\varvec{\epsilon }({\textbf{v}})) + (\lambda \nabla \hspace{-1.38885pt}\cdot \hspace{-1.38885pt}{{\textbf{W}}}, \nabla \hspace{-1.38885pt}\cdot \hspace{-1.38885pt}{{\textbf{v}}}) \\&\quad + (\lambda \delta _2 \nabla \hspace{-1.38885pt}\cdot \hspace{-1.38885pt}{{\textbf{u}}}, \nabla \hspace{-1.38885pt}\cdot \hspace{-1.38885pt}{{\textbf{v}}}) + ({\textbf{D}} \nabla \Psi , \nabla \psi ) - (\Psi , \gamma \nabla \hspace{-1.38885pt}\cdot \hspace{-1.38885pt}{{\textbf{v}}}) + (\gamma \nabla \hspace{-1.38885pt}\cdot \hspace{-1.38885pt}{{{\textbf{u}}}}, \psi ) + (\gamma \tau _2 \nabla \hspace{-1.38885pt}\cdot \hspace{-1.38885pt}{\dot{{\textbf{u}}}}, \psi )\\&\quad = ({\textbf{F}}, {\textbf{v}}) + (G, \psi ). \end{aligned} \end{aligned}$$

Now, in (7) we take $({\textbf{v}}, \psi ) = (\tau _2 \dot{{\textbf{u}}}, \varphi )$ and multiply everything by $\tau _2$:

$$\begin{aligned} \begin{aligned} \dfrac{1}{2}\dfrac{\textrm{d}}{\textrm{d}t} \Big (\Vert \tau _2\sqrt{\rho }\dot{{\textbf{u}}}\Vert ^2 +&\Vert \tau _2 {\textbf{u}}\Vert ^2_{\textbf{V}} + \Vert \sqrt{\tau _2 \tau _1 d_0} \varphi \Vert ^2+ \Vert \sqrt{\tau _2{\textbf{D}}} \nabla \Psi \Vert ^2\Big ) + \Vert \tau _2 \dot{{\textbf{u}}}\Vert ^2_{{\textbf{V}}\delta } + \Vert \sqrt{\tau _2 d_0} \varphi \Vert ^2 \\ =&- (\gamma \tau _2 \nabla \cdot {\textbf{u}},\varphi ) + (\tau _2^2{\textbf{f}},\dot{{\textbf{u}}}) + (\tau _2 G, \varphi ) \end{aligned} \end{aligned}$$

and in (8) we take $({\textbf{v}}, \psi ) =({\textbf{u}},\Psi )$:

$$\begin{aligned} \begin{aligned} \dfrac{1}{2}\dfrac{\textrm{d}}{\textrm{d}t} \Big (\Vert \sqrt{\rho }{\textbf{u}}\Vert ^2 +&\Vert {\textbf{W}}\Vert ^2_{\textbf{V}} + \Vert \sqrt{d_0} \Psi \Vert ^2 \Big ) + \Vert {\textbf{u}}\Vert ^2_{{\textbf{V}}\delta } + \Vert \sqrt{{\textbf{D}}}\nabla \Psi \Vert ^2 \\ =&(\gamma \tau _2 \nabla \hspace{-1.38885pt}\cdot \hspace{-1.38885pt}{{\textbf{u}}},\varphi ) - \dfrac{\textrm{d}}{\textrm{d}t}(\gamma \tau _2 \nabla \cdot {\textbf{u}},\Psi ) - (d_0 \tau _1 {\dot{\varphi }},\Psi ) + ({\textbf{F}}, {\textbf{u}}) + (G,\Psi ) \end{aligned} \end{aligned}$$

Summing up the two equations and integrating in time, we obtain:

$$\begin{aligned} \begin{aligned} \Vert \tau _2&\sqrt{\rho }\dot{{\textbf{u}}}\Vert ^2 + \Vert \sqrt{\rho }{\textbf{u}}\Vert ^2 + \Vert \tau _2 {\textbf{u}}\Vert ^2_{\textbf{V}} + \Vert {\textbf{W}}\Vert ^2_{\textbf{V}} + \Vert \sqrt{\tau _2 \tau _1 d_0} \varphi \Vert ^2+ \Vert \sqrt{\tau _2{\textbf{D}}} \nabla \Psi \Vert ^2 + \Vert \sqrt{d_0} \Psi \Vert ^2 \\ +&2\int _0^t \Vert {\textbf{u}}\Vert ^2_{{\textbf{V}}\delta } + 2\int _0^t \Vert \tau _2 \dot{{\textbf{u}}} \Vert ^2_{{\textbf{V}}\delta } +2 \int _0^t \Vert \sqrt{\tau _2 d_0} \varphi \Vert ^2 + 2\int _0^t\Vert \sqrt{{\textbf{D}}}\nabla \Psi \Vert ^2 + 2(\gamma \tau _2 \nabla \cdot {\textbf{u}},\Psi ) \\ +&2\int _0^t (d_0 \tau _1 {\dot{\varphi }},\Psi ) = I_0 + 2\int _0^t(\tau _2^2{\textbf{f}},\dot{{\textbf{u}}}) + 2\int _0^t(\tau _2 G, \varphi ) + 2\int _0^t ({\textbf{F}}, {\textbf{u}}) + 2\int _0^t (G,\Psi ), \end{aligned} \end{aligned}$$

where $I_0 = \Vert \tau _2\sqrt{\rho }\dot{{\textbf{u}}}_0\Vert ^2 + \Vert \sqrt{\rho }{\textbf{u}}_0\Vert ^2 + \Vert \tau _2 {\textbf{u}}_0\Vert ^2_{\textbf{V}} + \Vert \sqrt{\tau _2 \tau _1 d_0} \varphi _0\Vert ^2$. First, we treat the forcing terms using integration by parts in time and Poincaré and Korn inequalities [38]:

$$\begin{aligned} 2\int _0^t(\tau _2^2{\textbf{f}},\dot{{\textbf{u}}})&\lesssim \int _0^t \left\| \dfrac{\tau _2{\textbf{f}}}{\sqrt{\mu \delta _1}}\right\| ^2_{*} + \int _0^t \Vert \tau _2\dot{{\textbf{u}}}\Vert ^2_{{\textbf{V}}\delta }\\ 2\int _0^t(\tau _2 G, \varphi )&= - 2\int _0^t(\tau _2 g, \Psi ) + 2(\tau _2 G, \Psi ) \\&\lesssim \int _0^t \dfrac{\Vert \tau _2 g\Vert ^2_{*}}{\Vert \sqrt{{\textbf{D}}}\Vert ^2} + \int _0^t \Vert \sqrt{{\textbf{D}}}\nabla \Psi \Vert ^2 + \dfrac{\left\| \sqrt{\tau _2} G\right\| ^2_{*}}{{\Vert \sqrt{{\textbf{D}}}\Vert ^2}}+ \Vert \sqrt{\tau _2{\textbf{D}}}\nabla \Psi \Vert ^2 \\ 2\int _0^t ({\textbf{F}},{\textbf{u}})&\lesssim \int _0^t \left\| \dfrac{{\textbf{F}}}{\sqrt{\mu \delta _1}}\right\| ^2_{*} + \int _0^t \Vert {\textbf{u}}\Vert ^2_{{\textbf{V}}\delta }\\ 2\int _0^t (G,\Psi )&\lesssim \int _0^t \dfrac{\Vert G\Vert ^2_{*}}{\Vert \sqrt{{\textbf{D}}}\Vert ^2} + \int _0^t \Vert \sqrt{{\textbf{D}}}\nabla \Psi \Vert ^2. \end{aligned}$$

Then, we obtain:

9 $$\begin{aligned} \begin{aligned} \Vert \tau _2&\sqrt{\rho }\dot{{\textbf{u}}}\Vert ^2 + \Vert \sqrt{\rho }{\textbf{u}}\Vert ^2 + \Vert \tau _2 {\textbf{u}}\Vert ^2_{\textbf{V}} + \Vert {\textbf{W}}\Vert ^2_{\textbf{V}} + \Vert \sqrt{\tau _2 \tau _1 d_0} \varphi \Vert ^2 + \Vert \sqrt{d_0} \Psi \Vert ^2 + \int _0^t \Vert {\textbf{u}}\Vert ^2_{{\textbf{V}}\delta } \\ +&\int _0^t \Vert \tau _2 \dot{{\textbf{u}}} \Vert ^2_{{\textbf{V}}\delta } + 2 \int _0^t \Vert \sqrt{\tau _2 d_0} \varphi \Vert ^2 + 2(\gamma \tau _2 \nabla \cdot {\textbf{u}},\Psi ) + 2\int _0^t (d_0 \tau _1 {\dot{\varphi }},\Psi ) \lesssim I_0 \\ +&\dfrac{\left\| \sqrt{\tau _2} G\right\| ^2_{*}}{{\Vert \sqrt{{\textbf{D}}}\Vert ^2}} + \int _0^t \left\| \dfrac{\tau _2{\textbf{f}}}{\sqrt{\mu \delta _1}}\right\| ^2_{*} + \int _0^t \dfrac{\Vert \tau _2 g\Vert ^2_{*}}{\Vert \sqrt{{\textbf{D}}}\Vert ^2} + \int _0^t \left\| \dfrac{{\textbf{F}}}{\sqrt{\mu \delta _1}}\right\| ^2_{*} + \int _0^t \dfrac{\Vert G\Vert ^2_{*}}{\Vert \sqrt{{\textbf{D}}}\Vert ^2}. \end{aligned} \end{aligned}$$

We are left to control the tenth and eleventh terms on the left-hand side of (9). Concerning the first of the two, under the assumption $\tau _2\ge \tau _1$:

$$\begin{aligned} 2\int _0^t (d_0 \tau _1 {\dot{\varphi }},\Psi ) = \dfrac{\textrm{d}}{\textrm{d}t}\Vert \sqrt{\tau _1 d_0}\Psi \Vert ^2 - 2\int _0^t \Vert \sqrt{\tau _1 d_0} \varphi \Vert ^2 \end{aligned}$$

For the second one, we test problem (6) by $(\tau _2{\textbf{u}},0)$ and we get:

$$\begin{aligned} \dfrac{1}{2}\dfrac{\textrm{d}}{\textrm{d}t} \left( \Vert \sqrt{\tau _2\rho }{\textbf{u}}\Vert ^2 + \Vert \sqrt{\tau _2}{\textbf{W}}\Vert ^2_{\textbf{V}} \right) + \Vert \sqrt{\tau _2}{\textbf{u}}\Vert ^2_{{\textbf{V}}\delta } = (\gamma \tau _2 \nabla \cdot {\textbf{u}},\Psi ) + (\tau _2{\textbf{F}}, {\textbf{u}}) \end{aligned}$$

Finally, by substituting the two quantities into (9), we obtain that:

$$\begin{aligned} \dfrac{\textrm{d}}{\textrm{d}t}(\Vert&\sqrt{\tau _2\rho }{\textbf{u}}\Vert ^2 + \Vert \sqrt{\tau _2}{\textbf{W}}\Vert ^2_{\textbf{V}}+ \Vert \sqrt{\tau _1 d_0}\Psi \Vert ^2) + \Vert \tau _2\sqrt{\rho }\dot{{\textbf{u}}}\Vert ^2 + \Vert \sqrt{\rho }{\textbf{u}}\Vert ^2 + \left\| \dfrac{\tau _2}{2} {\textbf{u}}\right\| ^2_{\textbf{V}} + \Vert \sqrt{\tau _2}{\textbf{u}}\Vert ^2_{{\textbf{V}}\delta } \\ +&\Vert {\textbf{W}}\Vert ^2_{\textbf{V}} + \Vert \sqrt{\tau _2 \tau _1 d_0} \varphi \Vert ^2 + \Vert \sqrt{d_0} \Psi \Vert ^2 + \int _0^t \Vert {\textbf{u}} \Vert ^2_{{\textbf{V}}\delta } + \int _0^t \Vert \tau _2 \dot{{\textbf{u}}} \Vert ^2_{{\textbf{V}}\delta } + 2 \int _0^t \Vert \sqrt{(\tau _2-\tau _1) d_0} \varphi \Vert ^2 \\ \lesssim&\Vert {\textbf{F}}\Vert ^2_{*} + \dfrac{\left\| \sqrt{\tau _2} G\right\| ^2_{*}}{{\Vert \sqrt{{\textbf{D}}}\Vert ^2}} + \int _0^t \left\| \dfrac{\tau _2{\textbf{f}}}{\sqrt{\mu \delta _1}}\right\| ^2_{*} + \int _0^t \dfrac{\Vert \tau _2 g\Vert ^2_{*}}{\Vert \sqrt{{\textbf{D}}}\Vert ^2} + \int _0^t \left\| \dfrac{{\textbf{F}}}{\sqrt{\mu \delta _1}}\right\| ^2_{*} + \int _0^t \dfrac{\Vert G\Vert ^2_{*}}{\Vert \sqrt{{\textbf{D}}}\Vert ^2}. \end{aligned}$$

After a final integration in time and neglecting the positive integrals on the introduced auxiliary variables, we obtain the thesis. $\square$

### Remark 1

Theorem 1 provides two stability controls in $L^\infty ((0,T_f],{\textbf{L}}^2(\Omega ))$ and $L^2((0,T_f], {\textbf{H}}^1(\Omega ))$ on the displacement solution ${\textbf{u}}$. Concerning $\varphi$, we proved a control in $L^2((0,T_f], L^2(\Omega ))$. Moreover, a final control for $\dot{{\textbf{u}}}$ is provided in $L^2((0,T_f],{\textbf{L}}^2(\Omega ))$.

### Remark 2

Theorem 1 provides a robust estimate with respect to the quasi-static case ($\rho =0$). Moreover, the elastic case $\delta _1=\delta _2=0$ is simple to be obtained by changing the control on the forcing term using Grönwall lemma [54]. We observe that the estimate is not robust with respect to the conductivity ${\textbf{D}}$, the second Lamé parameter $\mu$, and the viscoelasticity retardation time $\delta _1$. However, in Section 5.3 we show that the proposed scheme is numerically robust with respect to the cases ${\textbf{D}}, \mu , \delta _1 \ll 1$.

## Polytopal Discontinuous Galerkin Discretization

This section aims to derive the fully-discrete scheme of problem (1). After introducing some preliminary concepts and assumptions about PolyDG methods, we detail the spatial discretization obtained via the PolyDG approximation, cf. Section 4.2. Then, for the time-integration of this problem, we consider two different cases, depending on the value of the time-relaxation parameter $\tau _1$. When $\tau _1 > 0$, both the equations in (1) are second-order hyperbolic, so we integrate in time with a Newmark-$\beta$ scheme. When $\tau _1 = 0$, equation (1a) is second-order hyperbolic, while equation (1b) is parabolic, then we couple an implicit Newmark-$\beta$ scheme for (1a), with a $\theta$-method for (1b), cf. Section 4.4.

### Preliminaries

First, we present the mesh assumptions, the discrete spaces, and some instrumental results for designing and analyzing PolyDG schemes. We introduce a subdivision ${\mathscr {T}}_h$ of the computational domain $\Omega$, whose elements are polygons/polyhedra in dimension $d = 2, 3$, respectively. Next, we define the interfaces (or internal faces) as subsets of the intersection of any two neighboring elements of ${\mathscr {T}}_h$. If $d=2$, an interface is a line segment, while if $d=3$, an interface is a planar polygon that we assume can be further decomposed into a set of triangles. The same holds for the boundary faces collected in the set ${\mathscr {F}}_B$, which yields a simplicial subdivision of $\partial \Omega$. Accordingly, we define ${\mathscr {F}}_I$ to be the set of internal faces and the set of all the faces as ${\mathscr {F}}_h={\mathscr {F}}_B\cup {\mathscr {F}}_I$.

As a basis for constructing the PolyDG approximation, we define fully-discontinuous polynomial spaces on the mesh ${\mathscr {T}}_h$. Given an element-wise constant polynomial degree $\ell :{\mathscr {T}}_h\rightarrow {\mathbb {N}}_{>0}$, which determines the order of the approximation, the discrete spaces are defined such as

$$\begin{aligned} \begin{aligned} V_h^{\ell }&= \left\{ v_h \in L^2(\Omega ) : v_h |_{\kappa } \in {\mathbb {P}}^{\ell _{\kappa }}(\kappa ) \ \ \forall \kappa \in {\mathscr {T}}_h \right\} , \quad {\textbf{V}}_h^{\ell } = \left[ V_h^{\ell } \right] ^d.\\ \end{aligned} \end{aligned}$$

where, for each $\kappa \in {\mathscr {T}}_h$, the space ${\mathbb {P}}^{\ell _{\kappa }}(\kappa )$ is spanned by polynomials of maximum degree $\ell _{\kappa }=\ell _{|\kappa }$. To analyze the convergence of the spatial discretization, we consider a mesh sequence $\{{\mathscr {T}}_h\}_{h\rightarrow 0}$ satisfying the following properties:

#### Assumption 2

The mesh sequence $\{{\mathscr {T}}_h\}_h$ satisfies the following properties [37]:

1. Shape Regularity: $$\forall K\in {\mathscr {T}}_h\;it\;holds: c_1 h_K^d\lesssim |K|\lesssim c_2h_K^d$$.
2. Contact Regularity: $\forall F\in {\mathscr {F}}$ with $F\subseteq {\overline{K}}$ for some $K\in {\mathscr {T}}_h$, it holds $h_K^{d-1}\lesssim |F|$, where |*F*| is the Hausdorff measure of the face *F*.
3. Submesh Condition: There exists a shape-regular, conforming, matching simplicial submesh $\widetilde{{\mathscr {T}}_h}$ conditions such that A.3

(*i*): $$\forall {\widetilde{K}}\in \widetilde{{\mathscr {T}}_h}\;\quad \exists K\in {\mathscr {T}}_h$$ such that $${\widetilde{K}}\subseteq K$$.
(*ii*): The family $\{\widetilde{{\mathscr {T}}_h}\}_h$ is shape and contact regular.
(*iii*): $\forall {\widetilde{K}}\in \widetilde{{\mathscr {T}}_h}, K\in {\mathscr {T}}_h$ with ${\widetilde{K}} \subseteq K$, it holds $h_K \lesssim h_{{\widetilde{K}}}$.

We remark that under A.1 the following inequality (called *discrete trace-inverse inequality*) holds (cf. [37] for all the details):

$$\begin{aligned} | v |_{L^2(\partial \kappa )} \lesssim \frac{\ell _{\kappa }}{h_{\kappa }^{1/2}} |v|_{L^2(\kappa )} \quad \forall v \in {\mathbb {P}}^{\ell _{\kappa }}(\kappa ), \end{aligned}$$

where the hidden constant is independent of $\ell _{\kappa }, h_{\kappa }$, and the number of faces per element. In the following discussion, we introduce the Weighted Symmetric Interior Penalty method (WSIP) [39]. The key ingredient of this method is to exploit weighted averages instead of the arithmetic ones used in the standard Interior Penalty formulation, cf. [6, 66]. The use of weighted averages was first introduced for elliptic problems in [63] and then developed for dG methods for dealing with advection-diffusion problems with locally vanishing diffusion [39]. In [20], the PolyDG-WSIP discretization of a thermo-hydromechanics problem is presented.

For the definition of the PolyDG-WSIP method, we introduce the weight function $\omega ^+:{\mathscr {F}}_I\rightarrow [0,1]$ [45]. Given an interior face $F \in {\mathscr {F}}_I$, we denote the values taken by $\omega ^+$ and $\omega ^- = 1-\omega ^+$ on the face *F* as $\omega |_F^{+}$ and $\omega |_F^{-}$, respectively. Given the function $\omega$, we can introduce the notion of weighted averages and jump operators, denoted with $\{ \hspace{-2.77771pt}\{\cdot \} \hspace{-2.77771pt}\}_{\omega }$ and $\left[ \hspace{-0.83328pt}\left[ \cdot \right] \hspace{-0.83328pt}\right]$, and normal jump, denoted with $\left[ \hspace{-0.83328pt}\left[ \cdot \right] \hspace{-0.83328pt}\right] _n$ [7, 39]:

$$\begin{aligned} \begin{aligned}&\left[ \hspace{-0.83328pt}\left[ a\right] \hspace{-0.83328pt}\right] = a^+ \mathbf {n^+} + a^- \mathbf {n^-}, \quad  &   \left[ \hspace{-0.83328pt}\left[ {\textbf{a}}\right] \hspace{-0.83328pt}\right] = {\textbf{a}}^+ \odot \mathbf {n^+} + {\textbf{a}}^- \odot \mathbf {n^-}, \quad  &   \left[ \hspace{-0.83328pt}\left[ {\textbf{a}}\right] \hspace{-0.83328pt}\right] _n = {\textbf{a}}^+ \cdot \mathbf {n^+} + {\textbf{a}}^- \cdot \mathbf {n^-}, \\&\{ \hspace{-2.77771pt}\{a \} \hspace{-2.77771pt}\}_{\omega } = \omega ^+ a^+ + \omega ^- a^, \quad  &   \{ \hspace{-2.77771pt}\{{\textbf{a}} \} \hspace{-2.77771pt}\}_{\omega } = \omega ^+ {\textbf{a}}^+ + \omega ^- {\textbf{a}}^-, \quad  &   \{ \hspace{-2.77771pt}\{{\textbf{A}} \} \hspace{-2.77771pt}\}_{\omega } = \omega ^+ {\textbf{A}}^+ + \omega ^- {\textbf{A}}^-, \end{aligned} \end{aligned}$$

where ${\textbf{a}} \odot {\textbf{n}} = {\textbf{a}}{\textbf{n}}^T$ and $a, \ {\textbf{a}}, \ {\textbf{A}}$ are (regular enough) scalar-, vector-, and tensor-valued functions, respectively. The notation $(\cdot )^{\pm }$ is used for the trace on *F* taken within the interior of $\kappa ^\pm$ and ${\textbf{n}}^\pm$ is the outer unit normal vector to $\partial \kappa ^\pm$. When the subscript $\omega$ is omitted, we consider $\omega ^+ = \omega ^- = 1/2$. Accordingly, on boundary faces $F\in {\mathscr {F}}_B$, we set

$$\begin{aligned} \left[ \hspace{-0.83328pt}\left[ a\right] \hspace{-0.83328pt}\right] = a {\textbf{n}},\ \ \{ \hspace{-2.77771pt}\{a \} \hspace{-2.77771pt}\}_{\omega } = a,\ \ \left[ \hspace{-0.83328pt}\left[ {\textbf{a}}\right] \hspace{-0.83328pt}\right] = {\textbf{a}} \odot {\textbf{n}},\ \ \{ \hspace{-2.77771pt}\{{\textbf{a}} \} \hspace{-2.77771pt}\}_{\omega } = {\textbf{a}},\ \ \left[ \hspace{-0.83328pt}\left[ {\textbf{a}}\right] \hspace{-0.83328pt}\right] _n = {\textbf{a}} \cdot {\textbf{n}},\ \ \{ \hspace{-2.77771pt}\{{\textbf{A}} \} \hspace{-2.77771pt}\}_{\omega } = {\textbf{A}}, \end{aligned}$$

for the averages, this corresponds to consider $\omega ^\pm$ single-valued and equal to 1.

From now on, for the sake of simplicity, we assume that the model parameters are element-wise constant. Moreover, for later use, we can introduce the quantities

$$\begin{aligned} \mu _{\kappa } = \mu |_{\kappa }, \quad \lambda _{\kappa } = \lambda |_{\kappa }, \quad \text {and } \overline{{\textbf{D}}}_{\kappa } = |\sqrt{\mathbf {{\textbf{D}}}|_{\kappa }}|_2^2, \end{aligned}$$

where $|\cdot |_2$ denotes the $\ell ^2$-norm in ${\mathbb {R}}^{d \times d}$.

### Discontinuous Galerkin Semi-discrete Problem

The aim of this section is to introduce the PolyDG-WSIP approximation of problem (1) and to derive the stability estimate of the semi-discrete formulation. The PolyDG-WSIP semi-discretization of problem (3) reads: *for any *$t \in (0, T_f]$*, find *$({\textbf{u}}_h, \varphi _h)(t) \in {\textbf{V}}^{\ell }_h \times V^{\ell }_h$* such that *$\forall \, ({\textbf{v}}_h, \psi _h) \in {\textbf{V}}^{\ell }_h \times V^{\ell }_h$:

10 $$\begin{aligned} \begin{aligned} {\mathscr {M}}_{u}(\ddot{{\textbf{u}}}_h, {\textbf{v}}_h)&+ {\mathscr {M}}_{\varphi , \tau _1}(\ddot{\varphi }_h, \psi _h) + {\mathscr {C}}_{\tau _2,h}(\ddot{{\textbf{u}}}_h, \psi _h) + {\mathscr {A}}_{e,\delta _1,h}(\dot{{\textbf{u}}}_h, {\textbf{v}}_h) + {\mathscr {A}}_{\text {div},\delta _2,h}(\dot{{\textbf{u}}}_h, {\textbf{v}}_h) \\&+ {\mathscr {M}}_{\varphi }({\dot{\varphi }}_h, \psi _h) + {\mathscr {C}}_h(\dot{{\textbf{u}}}_h, \psi _h) + {\mathscr {A}}_{e,h}({\textbf{u}}_h, {\textbf{v}}_h) + {\mathscr {A}}_{\text {div},h}({\textbf{u}}_h, {\textbf{v}}_h) \\  &+ {\mathscr {A}}_{\varphi ,h}(\varphi _h, \psi _h) - {\mathscr {C}}_h({\textbf{v}}_h, \varphi _h) = ({\textbf{f}},{\textbf{v}}_h)+(g, \psi _h) \end{aligned} \end{aligned}$$

*supplemented by initial conditions *$({\textbf{u}}_{h,0}, \, \varphi _{h,0}, \, \dot{{\textbf{u}}}_{h,0}, \, {\dot{\varphi }}_{h,0})$ *that are fitting approximations of the initial conditions of problem* (1). The bilinear forms labelled with the subscript ${}_h$ appearing in (10) readHere, for all $a \in V_h^{\ell }$ and ${\textbf{a}}\in {\textbf{V}}_h^{\ell }$, $\nabla _h a$ and $\nabla _h \hspace{-1.38885pt}\cdot \hspace{-1.38885pt}{{\textbf{a}}}$ denote the broken differential operators whose restrictions to each element $\kappa \in {\mathscr {T}}_h$ are defined as $\nabla a_{|\kappa }$ and $\nabla \hspace{-1.38885pt}\cdot \hspace{-1.38885pt}{{\textbf{a}}}_{|\kappa }$, respectively. Then, the broken version of the strain tensor is defined as $\varvec{\epsilon }_h({\textbf{u}}) = \left( \nabla _h {\textbf{u}} + \nabla _h {\textbf{u}}^T\right) /2$. We set:

$$\begin{aligned} \omega _{\mu }^{\pm } = \frac{\mu ^{\mp }}{\mu ^{+} + \mu ^{-}}, \quad \omega _{\delta _1}^{\pm } = \frac{(\mu \delta _1)^{\mp }}{(\mu \delta _1)^{+} + (\mu \delta _1)^{-}}, \quad \omega _{\lambda }^{\pm } = \frac{\lambda ^{\mp }}{\lambda ^{+} + \lambda ^{-}}, \\ \omega _{\delta _2}^{\pm } = \frac{(\lambda \delta _2)^{\mp }}{(\lambda \delta _2)^{+} + (\lambda \delta _2)^{-}},\quad \omega _{{\textbf{D}}}^{\pm } = \frac{\eta _{{\textbf{D}}_n}^{\mp }}{\eta _{{\textbf{D}}_n}^{+} + \eta _{{\textbf{D}}_n}^{-}} \end{aligned}$$

where $\eta _{{\textbf{D}}_n}^{\pm } = {\textbf{n}}^{{\pm }^T} \, {\textbf{D}}^{\pm } \, {\textbf{n}}^{{\pm }}$. The PolyDG-WSIP method requires the definition of the following stabilization functions $\sigma , \sigma _{\delta _1}, \xi , \xi _{\delta _2}, \zeta \in L^{\infty }({\mathscr {F}}_h)$ are defined according to [39] as:

11 $$\begin{aligned} \begin{aligned} \sigma&= \left\{ \begin{aligned}&\alpha _1 \gamma _{\mu } \underset{\kappa \in \{\kappa ^+,\kappa ^-\}}{\text{ max }}\left( \frac{\ell _{\kappa }^2}{h_{\kappa }}\right) \  &F \in {\mathscr {F}}_I,\\&\alpha _1 \mu _{\kappa } \ell _\kappa ^2 h_{\kappa }^{-1} \  &F \in {\mathscr {F}}_B,\\ \end{aligned} \right. \ \ \sigma _{\delta _1} =  &   \left\{ \begin{aligned}&\alpha _2 \gamma _{\delta _1} \underset{\kappa \in \{\kappa ^+,\kappa ^-\}}{\text{ max }}\left( \frac{\ell _{\kappa }^2}{h_{\kappa }}\right) \  &F \in {\mathscr {F}}_I,\\&\alpha _2 \mu _{\kappa } \delta _{1_{\kappa }} \ell _\kappa ^2 h_{\kappa }^{-1} \  &F \in {\mathscr {F}}_B,\\ \end{aligned} \right. \\ \xi&= \left\{ \begin{aligned}&\alpha _3 \gamma _{\lambda } \underset{\kappa \in \{\kappa ^+,\kappa ^-\}}{\text{ max }}\left( \frac{\ell _\kappa ^2}{h_{\kappa }}\right) \  &F \in {\mathscr {F}}_I,\\&\alpha _3 \lambda _{\kappa } \ell _\kappa ^2 h_{\kappa }^{-1} \  &F \in {\mathscr {F}}_B,\\ \end{aligned} \right. \ \ \xi _{\delta _2} =  &   \left\{ \begin{aligned}&\alpha _4 \gamma _{\delta _2} \underset{\kappa \in \{\kappa ^+,\kappa ^-\}}{\text{ max }}\left( \frac{\ell _\kappa ^2}{h_{\kappa }}\right) \  &F \in {\mathscr {F}}_I,\\&\alpha _4 \lambda _{\kappa } \delta _{2_{\kappa }} \ell _\kappa ^2 h_{\kappa }^{-1} \  &F \in {\mathscr {F}}_B,\\ \end{aligned} \right. \\ \zeta&= \left\{ \begin{aligned}&\alpha _5 \gamma _{{\textbf{D}}} \underset{\kappa \in \{\kappa ^+,\kappa ^-\}}{\text{ max }}\left( \frac{\ell _\kappa ^2}{h_{\kappa }}\right) \  &F \in {\mathscr {F}}_I,\\&\alpha _5 {\overline{{\textbf{D}}}_{\kappa }}^{-1} \ell _\kappa ^2 h_{\kappa }^{-1} \  &F \in {\mathscr {F}}_B,\\ \end{aligned} \right. \end{aligned} \nonumber \\ \end{aligned}$$

where $\alpha _1, \alpha _2, \alpha _3, \alpha _4,\alpha _5 \in {\mathbb {R}}$ are positive constants to be properly defined, $\ell _{\kappa }$ is the (local) polynomial degree of approximation, $h_{\kappa }$ is the diameter of the element $\kappa \in {\mathscr {T}}_h$, and the coefficients $\gamma _{\mu }$, $\gamma _{\delta _1}$, $\gamma _{\lambda }$, $\gamma _{\delta _2}$, and $\gamma _{{\textbf{D}}}$ are given by:

$$\begin{aligned}  &   \gamma _{\mu }^{\pm } = \frac{\mu ^{+} \, \mu ^{-}}{\mu ^{+} + \mu ^{-}}, \quad \gamma _{\delta _1}^{\pm } = \frac{(\mu \delta _1)^{+} \, (\mu \delta _1)^{-}}{(\mu \delta _1)^{+} + (\mu \delta _1)^{-}}, \, \\  &   \gamma _{\lambda }^{\pm } = \frac{\lambda ^{+} \, \lambda ^{-}}{\lambda ^{+} + \lambda ^{-}}, \quad \gamma _{\delta _2}^{\pm } = \frac{(\lambda \delta _2)^{+} \, (\lambda \delta _2)^{-}}{(\lambda \delta _2)^{+} + (\lambda \delta _2)^{-}}, \quad \gamma _{{\textbf{D}}} = \frac{\eta _{{\textbf{D}}_n}^{+} \, \eta _{{\textbf{D}}_n}^{-}}{\eta _{{\textbf{D}}_n}^{+} + \eta _{{\textbf{D}}_n}^{-}}. \end{aligned}$$

### *A-priori* Analysis of Semi-discrete Formulation

Before writing the stability theorem in the semi-discrete version, we introduce the following auxiliary dG norms $\forall {\textbf{u}} \in {\textbf{V}}_h^{\ell }$, $\forall \varphi \in V_h^{\ell }$:

$$\begin{aligned} \Vert {\textbf{u}}_h\Vert _\textrm{dG,e}^2 =&\,\Vert \sqrt{2\mu }\epsilon _h({\textbf{u}}_h)\Vert ^2+\Vert \sqrt{\lambda }\nabla _h \hspace{-1.38885pt}\cdot \hspace{-1.38885pt}{{\textbf{u}}_h}\Vert ^2 + \sum _{F\in {\mathscr {F}}} (\Vert \sqrt{\sigma }\left[ \hspace{-0.83328pt}\left[ {\textbf{u}}_h\right] \hspace{-0.83328pt}\right] \Vert ^2_F + \Vert \sqrt{\xi }\left[ \hspace{-0.83328pt}\left[ {\textbf{u}}_h\right] \hspace{-0.83328pt}\right] _{\textbf{n}}\Vert ^2_F), \\ \Vert {\textbf{u}}\Vert _{\textrm{dG},\delta }^2 =&\,\Vert \sqrt{2\mu \delta _1}\epsilon _h({\textbf{u}}_h)\Vert ^2+\Vert \sqrt{\lambda \delta _2}\nabla _h \hspace{-1.38885pt}\cdot \hspace{-1.38885pt}{{\textbf{u}}_h}\Vert ^2 + \sum _{F\in {\mathscr {F}}} (\Vert \sqrt{\sigma _{\delta _1}}\left[ \hspace{-0.83328pt}\left[ {\textbf{u}}_h\right] \hspace{-0.83328pt}\right] \Vert ^2_F + \Vert \sqrt{\xi _{\delta _2}}\left[ \hspace{-0.83328pt}\left[ {\textbf{u}}_h\right] \hspace{-0.83328pt}\right] _{\textbf{n}}\Vert ^2_F), \\ \Vert \varphi _h\Vert _{\textrm{dG},\varphi }^2 =&\, \Vert \sqrt{{\textbf{D}}}\nabla _h\varphi _h\Vert ^2 + \sum _{F\in {\mathscr {F}}} \Vert \sqrt{\zeta }\left[ \hspace{-0.83328pt}\left[ \varphi _h\right] \hspace{-0.83328pt}\right] \Vert ^2_F. \end{aligned}$$

Moreover, we introduce the additional dG norms to derive the convergence estimate $\forall {\textbf{u}} \in {\textbf{H}}^{\ell }({\mathscr {T}}_h)$, $\forall \varphi \in H^{\ell }({\mathscr {T}}_h)$:

$$\begin{aligned} |\!\!\,|\!\!\,|{\textbf{u}}_h|\!\!\,|\!\!\,|_\textrm{dG,e}^2 =&\,\Vert {\textbf{u}}_h\Vert _\textrm{dG,e}^2 + \sum _{F\in {\mathscr {F}}} \Vert \{ \hspace{-2.77771pt}\{\sqrt{2\mu }\mathbf {\epsilon }_h({\textbf{u}}_h) \} \hspace{-2.77771pt}\}_{\omega _{\mu }}\Vert ^2_F, \\ |\!\!\,|\!\!\,|{\textbf{u}}_h|\!\!\,|\!\!\,|_\mathrm {dG,\delta }^2 =&\, \Vert {\textbf{u}}_h\Vert _\mathrm {dG,\delta }^2 + \sum _{F\in {\mathscr {F}}} \Vert \{ \hspace{-2.77771pt}\{\sqrt{2\mu \delta _1}\mathbf {\epsilon }_h({\textbf{u}}_h) \} \hspace{-2.77771pt}\}_{\omega _{\delta _1}}\Vert ^2_F, \\ |\!\!\,|\!\!\,|\varphi _h|\!\!\,|\!\!\,|_{\textrm{dG},\varphi }^2 =&\,\Vert \varphi _h\Vert _{\textrm{dG},\varphi }^2 + \sum _{F\in {\mathscr {F}}} \Vert \{ \hspace{-2.77771pt}\{{\textbf{D}}\nabla _h\varphi _h \} \hspace{-2.77771pt}\}_{\omega _{\textbf{D}}}\Vert ^2_F. \end{aligned}$$

#### Theorem 2

(Stability estimate) Let us consider – for any time $t\in (0,T_f]$ – $({\textbf{u}}_h,\varphi _h)(t)\in {\textbf{V}}_h^\ell \times V^\ell _h$ to be the solution of the semi-discrete problem (10) with homogeneous Dirichlet boundary conditions. Under Assumptions 1,  2, and assuming the following additional regularity on the forcing terms ${\textbf{f}}\in C^0(0,T_f; {\textbf{L}}^2(\Omega ))$, $g\in C^0(0,T_f; L^2(\Omega ))$, the following stability estimate holds:

$$\begin{aligned}&\Vert \sqrt{\tau _2\rho }{\textbf{u}}_h\Vert ^2 + \int _0^t \left( \Vert \tau _2\sqrt{\rho }\dot{{\textbf{u}}}_h\Vert ^2 + \Vert \sqrt{\rho }{\textbf{u}}_h\Vert ^2 + \left\| \dfrac{\tau _2}{2} {\textbf{u}}_h\right\| ^2_\textrm{dG,e} + \Vert \tau _2{\textbf{u}}_h\Vert ^2_{\textrm{dG},\delta } + \Vert \sqrt{\tau _2 \tau _1 d_0} \varphi _h\Vert ^2 \right) \lesssim \\  &\Vert \sqrt{\tau _2\rho }{\textbf{u}}_{h,0}\Vert ^2 + t I_{h,0} + \int _0^t \left( \Vert {\textbf{F}}\Vert ^2 + \dfrac{\left\| \sqrt{\tau _2} G\right\| ^2}{{\Vert \sqrt{{\textbf{D}}}\Vert ^2}} + t\left\| \dfrac{\tau _2{\textbf{f}}}{\sqrt{\mu \delta _1}}\right\| ^2 \hspace{-4pt} + t\dfrac{\Vert \tau _2 g\Vert ^2}{\Vert \sqrt{{\textbf{D}}}\Vert ^2} + t\left\| \dfrac{{\textbf{F}}}{\sqrt{\mu \delta _1}}\right\| ^2 \hspace{-4pt} + t\dfrac{\Vert G\Vert ^2}{\Vert \sqrt{{\textbf{D}}}\Vert ^2}\right) \hspace{-2pt}, \end{aligned}$$

where:

$$\begin{aligned} \begin{aligned} {\textbf{F}} =&\,\int _0^t {\textbf{f}} + \rho \dot{{\textbf{u}}}_{h,0} - 2 \nabla \cdot \mu \delta _1\varvec{\epsilon }({\textbf{u}}_{h,0}) - \nabla (\lambda \delta _2\nabla \cdot {\textbf{u}}_{h,0}), \\ G =&\, \int _0^t g + d_0 (\varphi _{h,0} + \tau _1 {\dot{\varphi }}_{h,0}) + \gamma (\nabla \cdot {\textbf{u}}_{h,0}+\tau _2\nabla \cdot \dot{{\textbf{u}}}_{h,0}), \\ I_{h,0} =&\, \Vert \tau _2\sqrt{\rho }\dot{{\textbf{u}}}_{h,0}\Vert ^2 + \Vert \sqrt{\rho }{\textbf{u}}_{h,0}\Vert ^2 + \Vert \tau _2 {\textbf{u}}_{h,0}\Vert ^2_\textrm{dG} + \Vert \sqrt{\tau _2 \tau _1 d_0} \varphi _{h,0}\Vert ^2. \end{aligned} \end{aligned}$$

In this theorem, the (hidden) stability constant is independent of the physical parameters.

#### Proof

The proof of the stability can be adapted following the same steps of Theorem 1. The only differences are related to Poincaré and Korn’s inequalities, for which we use their discrete version (cf. [22, 37]). $\square$

To prove the *a-priori* error estimate we introduce the interpolants of the solutions ${\textbf{u}}_\textrm{I}\in {\textbf{V}}_h^\ell$ and $\varphi _\textrm{I}\in V_h^\ell$ [9].

#### Proposition 1

Let Assumption 2 be fulfilled. If $d \ge 2$, then the following estimates hold:

$$\begin{aligned}  &   \forall {\textbf{u}}\in {\textbf{H}}^n({\mathscr {T}}_h),\; \exists {\textbf{u}}_\textrm{I}\in {\textbf{V}}_h^\ell : \; \Vert {\textbf{u}}-{\textbf{u}}_\textrm{I}\Vert ^2 + |\!\!\,|\!\!\,|{\textbf{u}}-{\textbf{u}}_\textrm{I}|\!\!\,|\!\!\,|_\textrm{dG,e}^2 \lesssim \sum _{K\in {\mathscr {T}}_h} \dfrac{h_K^{s-2}}{\ell ^{2n-3}} \Vert {\textbf{u}}\Vert _{{\textbf{H}}^n(K)}^2, \\  &   \forall \varphi \in H^n({\mathscr {T}}_h),\; \exists \varphi _\textrm{I}\in V_h^\ell : \; \Vert \varphi -\varphi _\textrm{I}\Vert ^2 + |\!\!\,|\!\!\,|\varphi -\varphi _\textrm{I}|\!\!\,|\!\!\,|_\mathrm {dG,\varphi }^2 \lesssim \sum _{K\in {\mathscr {T}}_h} \dfrac{h_K^{s-2}}{\ell ^{2n-3}} \Vert \varphi \Vert _{H^n(K)}^2, \end{aligned}$$

where $$s= \min \{\ell +1,n\}$$.

#### Theorem 3

(*A-priori* error estimate) Let us consider – for any time $t\in (0,T_f]$ – $({\textbf{u}},\varphi )(t)\in {\textbf{V}}\times V$ and $({\textbf{u}}_h,\varphi _h)(t)\in {\textbf{V}}_h^\ell \times V_h^l$ to be the solution of the problems (3) and (10), respectively, with homogeneous Dirichlet boundary conditions, and sufficiently large penalty parameters. Under Assumptions 1, 2, then the following estimate holds:

$$\begin{aligned} \begin{aligned} \Vert {\textbf{u}}-{\textbf{u}}_h\Vert ^2 +&\int _0^t \left( \Vert \varphi -\varphi _h\Vert ^2 + \Vert \dot{{\textbf{u}}}-\dot{{\textbf{u}}}_h\Vert ^2 + \Vert {\textbf{u}}-{\textbf{u}}_h\Vert ^2 + \Vert {\textbf{u}}-{\textbf{u}}_h\Vert _\textrm{dG,e}^2 \right) \lesssim \\ (1 + t e^t)&\sum _{K\in {\mathscr {T}}_h} \dfrac{h_K^{s-2}}{\ell ^{2n-3}} \Big [\Vert {\textbf{u}}\Vert _{{\textbf{H}}^n(K)}^2 + \int _0^t\Big (\Vert \ddot{{\textbf{u}}}\Vert _{{\textbf{H}}^n(K)}^2 + \Vert \dot{{\textbf{u}}}\Vert _{{\textbf{H}}^n(K)}^2 + \Vert {\textbf{u}}\Vert _{{\textbf{H}}^n(K)}^2 \\&\qquad + \Vert {\textbf{W}}\Vert _{{\textbf{H}}^n(K)}^2 + \Vert {\dot{\varphi }}\Vert _{{H}^n(K)}^2 + \Vert \varphi \Vert _{{H}^n(K)}^2 + \Vert \Psi \Vert _{{H}^n(K)}^2 \Big )\Big ], \end{aligned} \end{aligned}$$

where $${\textbf{W}} = \int _0^t{\textbf{u}}(s)\textrm{d}s$$, and $$\Psi (t) = \int _0^t\varphi (s)\textrm{d}s$$.

#### Proof

The proof of Theorem 3 is reported in Appendix B. $\square$

#### Remark 3

In the statement of Theorem 3, we neglect the dependence on the physical parameters. However, in the proof reported in Appendix B, the result has the same robustness properties as Theorem 1, due to the use of the same inequalities in the proof of the stability estimate.

### Time Discretization

This section aims to introduce the time discretization of the semi-discrete problem (10). The time-integration scheme depends on the parameters we consider in (1). Namely, when $\tau _1 > 0$, the system is second-order hyperbolic, and we choose to use a Newmark-$\beta$ method for the whole system. When $\tau _1 = 0$, the second equation (1b) is parabolic, then we couple a Newmark-$\beta$ method for the first equation with a $\theta$-method for the second one. We denote by Newmark-$\beta$-$\theta$ the coupling of these two time-marching schemes.

By fixing a basis for the space ${\textbf{V}}_h^{\ell } \times V_h^{\ell }$ and denoting by $\left[ {\textbf{U}}, \varvec{\Phi } \right] ^T$ the vector of the expansion coefficients of the variables $({\textbf{u}}_h, \varphi _h)$, we can rewrite the semi-discrete problem (10) in the equivalent form:

12 $$\begin{aligned} \begin{aligned} \begin{bmatrix} {\textbf{M}}_{u} &  0 \\ {\textbf{C}}_{\tau _2} &  {\textbf{M}}_{\varphi , \tau _1} \end{bmatrix} \begin{bmatrix} \ddot{{\textbf{U}}} \\ \ddot{\varvec{\Phi }} \end{bmatrix}&+ \begin{bmatrix} {\textbf{A}}_{e,\delta _1} + {\textbf{A}}_{\text {div},\delta _2} &  0 \\ {\textbf{C}} &  {\textbf{M}}_{\varphi } \end{bmatrix} \begin{bmatrix} \dot{{\textbf{U}}} \\ \dot{\varvec{\Phi }} \end{bmatrix} + \begin{bmatrix} {\textbf{A}}_e + {\textbf{A}}_{\text {div}} &  - {\textbf{C}}^T \\ 0 &  {\textbf{A}}_{\varphi } \end{bmatrix} \begin{bmatrix} {\textbf{U}} \\ \varvec{\Phi } \end{bmatrix} = \begin{bmatrix} {\textbf{F}} \\ {\textbf{G}} \end{bmatrix} \end{aligned} \nonumber \\ \end{aligned}$$

with initial conditions ${\textbf{U}}(0) = {\textbf{U}}_0$, $\dot{{\textbf{U}}}(0) = {\textbf{U}}_1$, $\Phi (0) = \Phi _0$, and (when $\tau _1>0$) ${\dot{\Phi }}(0) = \Phi _1$. The vectors ${\textbf{F}}, {\textbf{G}}$ are representations of the linear functionals appearing on the right-hand side of (10).

To integrate (12) in time, we introduce a time-step $\Delta t = T_f/n$, with $n\in {\mathbb {N}}_{>0}$, discretize the interval $(0, T_f]$ as a sequence of time instants $\{ t_k \}_{0\le k\le n}$ such that $t_{k+1} - t_k = \Delta t$.

#### Case $\tau _1 > 0$ (Newmark-$\beta$ method)

We start by defining ${\textbf{X}}^k = {\textbf{X}}(t^k)$, with ${\textbf{X}} = \left[ {\textbf{U}}, \varvec{\Phi } \right] ^T$. Next, we rewrite (12) in a compact form as ${\textbf{A}} \ddot{{\textbf{X}}} + {\textbf{B}} \dot{{\textbf{X}}} + {\textbf{C}} {\textbf{X}} = {\textbf{F}}$ and derive

13 $$\begin{aligned} \ddot{{\textbf{X}}} = {\textbf{A}}^{-1} \left( {\textbf{F}} - {\textbf{B}} \dot{{\textbf{X}}} - {\textbf{C}} {\textbf{X}} \right) = {\textbf{A}}^{-1} {\textbf{F}} - {\textbf{A}}^{-1} {\textbf{B}} \dot{{\textbf{X}}} - {\textbf{A}}^{-1}{\textbf{C}} {\textbf{X}} = {\mathscr {L}}(t, {\textbf{X}}, \dot{{\textbf{X}}}). \end{aligned}$$

Last, we integrate in time (13) with the use of Newmark-$\beta$ scheme, that exploits a Taylor expansion for ${\textbf{X}}$ and ${\textbf{Y}} = \dot{{\textbf{X}}}$:

$$\begin{aligned} \left\{ \begin{aligned}&{\textbf{X}}^{k+1} = {\textbf{X}}^{k} + \Delta t {\textbf{Y}}^{k} + \Delta t^2 \left( \beta _N {\mathscr {L}}^{k+1} + (\frac{1}{2} - \beta _N) {\mathscr {L}}^{k} \right) , \\&{\textbf{Y}}^{k+1} = {\textbf{Y}}^{k} + \Delta t \left( \gamma _N {\mathscr {L}}^{k+1} + (1 - \gamma _N) {\mathscr {L}}^{k} \right) , \end{aligned} \right. \end{aligned}$$

where ${\mathscr {L}}^k = {\mathscr {L}}(t^k, {\textbf{X}}^k, \dot{{\textbf{X}}}^k)$ and the Newmark parameters $\beta _N, \gamma _N$ satisfy: $0 \le 2 \beta _N \le 1$, $0 \le \gamma _N \le 1$. The typical choices for the Newmark parameters, that ensure unconditional stability and second-order accuracy for the scheme, are $\beta _N = 1/4$ and $\gamma _N = 1/2$. These are the values used in all the numerical tests of Section 5.

#### Case $\tau _1 = 0$ (Coupling Newmark-$\beta$ and $\theta$-methods)

In this second case, we adopt a coupling between a Newmark-$\beta$ method for discretizing the first equation of (12) and a $\theta$-method for the pressure equation. The complete calculations are reported in the Appendix A. For the sake of clarity, we report the final formulation in a compact form:

14 $$\begin{aligned} \textbf{LX}^{n+1} = \textbf{RX}^{n} + {\textbf{S}}^{n+1} \qquad n>0, \end{aligned}$$

where:

$$\begin{aligned}&{\textbf{L}} = \begin{bmatrix} \frac{1}{\beta _N \Delta t^2} {\textbf{M}}_u + {\textbf{A}}_u + \frac{\gamma _N}{\beta _N \Delta t} {\textbf{A}}_{u,\delta } &  -{\textbf{C}}^T &  0 &  0 \\ {\textbf{C}}_u &  \frac{1}{\Delta t}{\textbf{M}}_{\varphi } + \theta {\textbf{A}}_{\varphi } &  0 &  0 \\ 0 &  0 &  {\textbf{I}} &  -\Delta t \gamma _N {\textbf{I}} \\ - \frac{1}{\beta _N \Delta t^2} {\textbf{I}} &  0 &  0 &  {\textbf{I}} \\ \end{bmatrix} \\&{\textbf{R}} = \begin{bmatrix} \frac{1}{\beta _N \Delta t^2} {\textbf{M}}_u + \frac{\gamma _N}{\beta _N \Delta t} {\textbf{A}}_{u,\delta } &  0 &  \frac{1}{\beta _N \Delta t} {\textbf{M}}_u - \frac{\beta _N-\gamma _N}{\beta _N} {\textbf{A}}_{u,\delta } &  \frac{1 - 2\beta _N}{2\beta _N} {\textbf{M}}_u - \frac{\Delta t (2\beta _N-\gamma _N)}{2\beta _N} {\textbf{A}}_{u,\delta } \\ {\textbf{C}}_u &  \frac{1}{\Delta t}{\textbf{M}}_{\varphi } - {\tilde{\theta }}{\textbf{A}}_{\varphi } &  {\textbf{C}}_z &  {\textbf{C}}_a \\ 0 &  0 &  {\textbf{I}} &  \Delta t (1-\gamma _N) {\textbf{I}} \\ - \frac{1}{\beta _N \Delta t^2} {\textbf{I}} &  0 &  - \frac{1}{\beta _N \Delta t} {\textbf{I}} &  \frac{2\beta _N - 1}{2 \beta _N}{\textbf{I}} \\ \end{bmatrix} \\&{\textbf{X}}^n = \begin{bmatrix} {\textbf{U}}^n, \ \varvec{\Phi }^n, \ {\textbf{Z}}^n, \ {\textbf{A}}^n \end{bmatrix}^T \qquad {\textbf{S}}^{n+1} = \begin{bmatrix} {\textbf{F}}^{n+1}, \ \theta {\textbf{G}}^{n+1} + {\tilde{\theta }} {\textbf{G}}^{n}, \ 0, \ 0 \end{bmatrix}^T \end{aligned}$$

##### Remark 4

From an operational point of view, we do not solve the whole system (14). For the sake of reducing the overall computational cost, we solve it just for ${\textbf{U}}^{n+1}$, $\varvec{\Phi }^{n+1}$. Then, we update the values of ${\textbf{A}}^{n+1}$ and ${\textbf{Z}}^{n+1}$.

## Numerical Results

This section aims to assess the performance of the proposed scheme in terms of accuracy and robustness with respect to the model parameters. Then, we test the method by addressing some benchmark and literature test cases. The numerical implementation is carried out in the open-source lymph library [5], implementing the PolyDG method for multiphysics. In all the tests, the PolyDG space discretization is solved monolithically, and it is coupled with the Newmark-$\beta$ method when $\tau _1 > 0$ and with a coupled Newmark-$\beta$-$\theta$-method when $\tau _1 = 0$ (cf. Section 4.4) for time integration. The parameters of the Newmark-$\beta$ and $\theta$-method are $\gamma _N = 1/2$, $\beta _N = 1/4$, and $\theta = 1/2$. All the penalty coefficients $\alpha _i$, $i=1,2,\dots ,5$ in (11) are set equal to 10.

### Convergence Tests

*Convergence vs space discretization parameters.* The aim of this section is to assess the performance of the proposed scheme in terms of accuracy with respect to the space discretization parameters, i.e., the mesh size *h* and the polynomial degree of approximation $\ell$.

**Table 2:** Convergence test: problem parameters for the convergence analysis

**Coefficient**	**Value**		**Coefficient**	**Value**
$\rho [\hbox {kg\,m}^{3}]$	1		$\gamma [-]$	1
$\mu \ [\hbox {Pa}]$	1		$\lambda [\hbox {Pa}]$	1
$d_0 \ [{\hbox {Pa}^{-1}}]$	1		${\textbf{D}} [\hbox {m}^{2}\,\hbox {Pa}^{-1}\,\hbox {s}^{-1}]$	${\textbf{I}}$
$\delta _1 [\hbox {s}]$	1		$\delta _2 [\hbox {s}]$	1
$\tau _1 [\hbox {s}]$	1		$\tau _2 [\hbox {s}]$	1

We consider problem (1) in the square domain $\Omega = (0,1)^2$ with manufactured analytical solutions:

$$\begin{aligned} \begin{aligned} {\textbf{u}} = \sin (2 \pi t) \left[ \begin{aligned} x^2 \sin (\pi x) \sin (\pi y) \\ - x^2 \sin (\pi x) \sin (\pi y) \end{aligned} \right] , \quad \varphi = \sin (\sqrt{2} \pi t) \left( x^2 \cos \left( \frac{\pi x}{2}\right) \sin (\pi x) \right) . \end{aligned} \end{aligned}$$

The initial and boundary conditions and the forcing terms are inferred from the exact solutions. The model coefficients are chosen as reported in Table 2. We remark that - for completeness - we also assess the method’s performance for the case $\tau _1 = 0$.

**Fig. 1:**
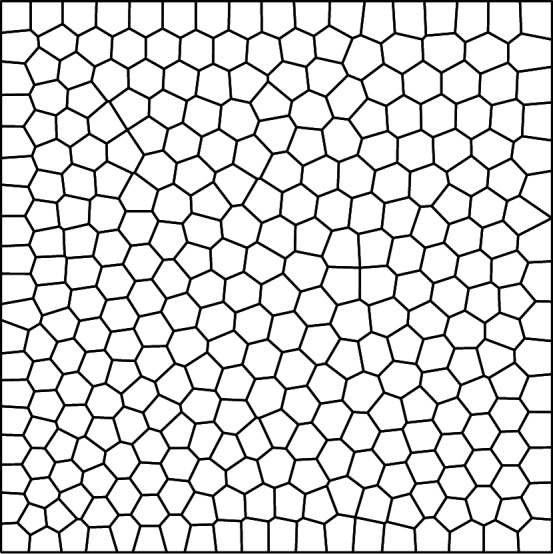
Convergence test: example of a 2D Voronoi polygonal mesh made of 300 elements

For the *h*-convergence, a sequence of polygonal meshes in Figure 1 is considered, and we consider polynomial degree $\ell = 3$. At the same time, for the $\ell$-convergence we fix a computational mesh of 100 elements and vary the polynomial degree $\ell = 1,2,\dots ,5$. The time discretization parameters are $T_f = 0.1$, $\Delta t ={5 \cdot 10^{-5}}$.

**Fig. 2:**
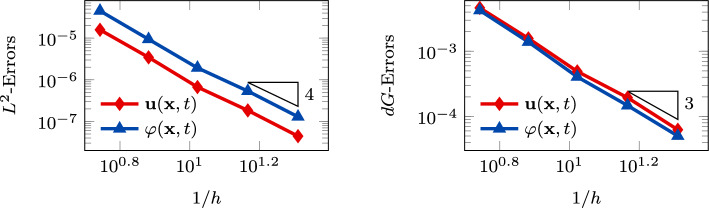
Convergence test vs *h* ($\tau _1 = 1 0$): computed errors in $L^2$-norm (left) and *dG*-norm (right) versus 1/*h* (*log-log* scale). The errors are computed at the final time $T_f$. The polynomial degree of approximation is taken as $\ell = 3$

**Fig. 3:**
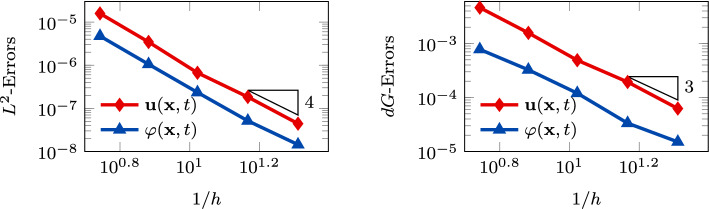
Convergence test vs h ($\tau _1 = 0$): computed errors in $L^2$-norm (left) and d*G*-norm (right) versus 1/h (loglog scale). The errors are computed at the final time $T_f$ . The polynomial degree of approximation is taken as $\ell = 3$

**Fig. 4:**
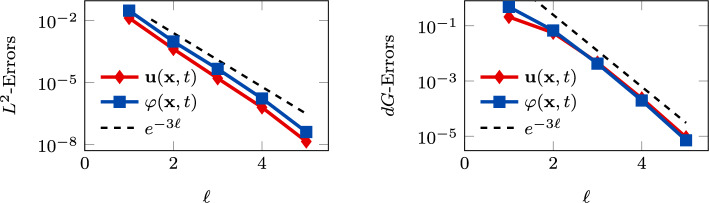
Convergence test vs $\ell$ ($\tau _1 = 1$): computed errors in $L^2$-norm (left) and *dG*-norm (right) versus (*semilog* scale). The errors are computed at the final time $T_f$ . The computational mesh is made of 100 polygons

In Figure 3, we report the $L^2$ and *dG*-errors for the two unknowns with respect to the mesh size (*log-log* scale). In agreement with the results expected by Theorem 3 (a-priori error estimate) and by the theory of the PolyDG methods (cf. [26, 27]), we observe that, as we are using $\ell = 3$, the *dG*-errors show a convergence rate proportional to $h^3$. Moreover, concerning the $L^2$-errors, we observe that the errors decay as $h^{\ell + 1}$. In Figure 4, we report the results for the same convergence test, but taking $\tau _1 = 0$ in (1b). Also, for this configuration, the numerical results match the predicted convergence rates of the PolyDG framework.

**Fig. 5:**
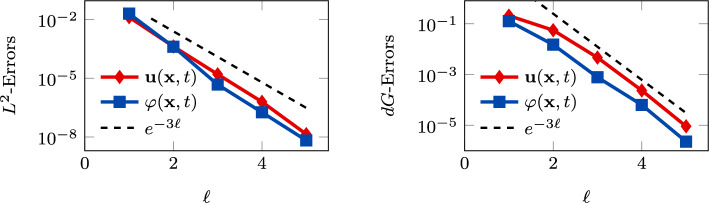
Convergence test vs $$\ell $$ ($$\tau _1 = 0$$): computed errors in $$L^2$$-norm (left) and *dG*-norm (right) versus (*semilog* scale). The errors are computed at the final time $$T_f $$. The computational mesh is made of 100 polygons

In Figure 4, Figure 5, we report the results for the $\ell$-convergence test. We observe that, for both the model configurations, the errors of the two variables decay exponentially.

*Convergence vs time discretization parameter.* This section aims to assess the performance of the proposed scheme in terms of accuracy with respect to the time-step $\Delta t$. To focus on the time-marching scheme, we consider problem (1) in the square domain $\Omega = (0,1)^2$ with manufactured analytical solutions

$$\begin{aligned} \begin{aligned} {\textbf{u}} = \sin (2 \pi t) \left[ \begin{aligned}&x + y \\&3x - 5y \end{aligned} \right] , \quad \varphi = \sin (\sqrt{2} \pi t) \left( 10x + 6y \right) . \end{aligned} \end{aligned}$$

The initial conditions, boundary conditions, and forcing terms are inferred from the exact solutions. The model coefficients are chosen as reported in Table 2. To test the convergence of the scheme with respect to $\Delta t$ (both for the Newmark-$\beta$ and for the coupled Newmark-$\beta$-$\theta$ method), we fix a mesh of $N = 100$ elements with $\ell = 1$, and we fix the final time $T_f = 0.1$. Then, we vary the time-step parameter.

**Fig. 6:**
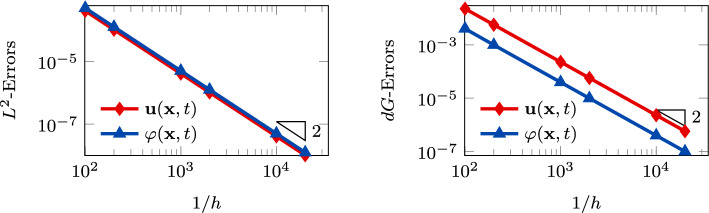
Convergence test vs $\Delta t$ *t* ($\tau _1 = 1$): computed errors in $L^2$-norm (left) and *dG*-norm (right) versus 1/*h* (*log*-*log* scale). The errors are computed at the final time $T_f$ . The time-marching scheme is the Newmark-$\beta$ method

**Fig. 7:**
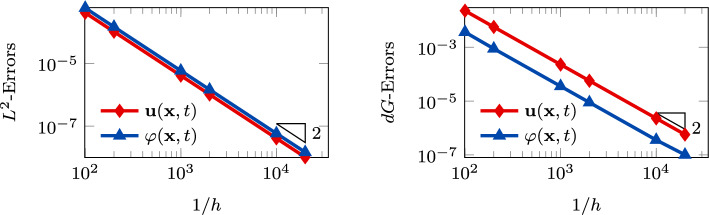
Convergence test vs $\Delta t$ *t* ($\tau _1 = 0$): computed errors in $L^2$-norm (left) and *dG*-norm (right) versus 1/*h* (*log*-*log* scale). The errors are computed at the final time $T_f$ . The time-marching scheme is the coupled Newmark-$\beta$ and $\theta$-method

In Figure  6, Figure 7 we report the results for the convergence against the time-step $\Delta t$. We observe that for both the Newmark-$\beta$ scheme ($\tau _1 \ne 0$) and for the coupled Newmark-$\beta$-$\theta$ time-marching schemes ($\tau _1 = 0$), we have second-order accuracy. The time-integration schemes’ parameters we used in this test are $\gamma _N = 1/2$, $\beta _N = 1/4$, and $\theta = 1/2$, as these choices ensure unconditional stability and second-order accuracy.

### Superconvergence Tests

The aim of this section is to prove that the PolyDG scheme proposed for this problem is not only optimal convergence, but it also shows some superconvergence properties. To this aim, we consider the following exact solutions:

$$\begin{aligned} \begin{aligned} {\textbf{u}}(x,y,t)&\ = \nu _u \, \sin (2 \pi t) \left( \begin{aligned}&x^2 \sin (\pi x) \sin (\pi y) \\&- x^2 \sin (\pi x) \sin (\pi y) \end{aligned} \right) , \\ \varphi (x,y,t)&\ = \nu _{\varphi } \, \sin (\sqrt{2} \pi t) \left( x^2 \cos \left( \frac{\pi x}{2}\right) \sin (\pi x) \right) , \end{aligned} \end{aligned}$$

from which we infer initial and boundary conditions, as well as forcing terms. The parameters $\nu _u$, $\nu _{\varphi }$ control the magnitude of the displacement and the generalized pressure, respectively. The model coefficients are reported in Table 2 (chosen as in the convergence test). We observe that the optimal convergence property has been already proven in Section 5.1 by setting $\nu _u = \nu _{\varphi } = 1$.

To observe the better robustness of the scheme with respect to large pressures, we propose two different tests. In the first, we set $\nu _u = 0.1$, $\nu _{\varphi } = 10^{4}$, we consider the same sequence of meshes as in Section 5.1 and polynomial degree $\ell = 2$. For the second test, we fix $\nu _u = 0.1$, the mesh, and the polynomial degree of approximation; then we vary the values of $\nu _{\varphi } = \left[ 1, 10^{1},10^{2},10^{3},10^{4},10^{5},10^{6}\right]$. We consider the following discretization parameters for the second test: $N = 400, \ \ell = 2$.

**Table 3:** Superconvergence test: computed errors and convergence rates in $L^2$- and *dG*-norms versus *h* using as polynomial degree of approximation $\ell = 2$

1/*h*	$\Vert {\textbf{e}}^{u}\Vert _{L^2}$	$\text {roc}^{u}_{L^2}$	$\Vert {\textbf{e}}^{u}\Vert _{dG}$	$\text {roc}^{u}_{dG}$	$\Vert e^{p}\Vert _{L^2}$	$\text {roc}^{p}_{L^2}$	$\Vert e^{p}\Vert _{dG}$	$\text {roc}^{p}_{dG}$
5.53	${4.49 \cdot 10^{-3}}$	-	0.15	-	8.47	-	1411.33	-
7.62	${1.06 \cdot 10^{-3}}$	4.49	0.06	3.11	2.83	3.42	779.86	1.85
10.53	${2.86 \cdot 10^{-4}}$	4.06	0.02	3.48	0.85	3.73	373.68	2.28
14.68	${9.30 \cdot 10^{-5}}$	3.38	${7.47 \cdot 10^{-3}}$	2.69	0.36	2.62	196.18	1.94
20.45	${3.12 \cdot 10^{-5}}$	3.30	${2.70 \cdot 10^{-3}}$	3.07	0.12	3.19	96.92	2.13

By looking at Table 3 we observe the superconvergence phenomenon for the displacement field. Indeed, we observe that using a polynomial degree of approximation equal to $\ell$, then the error of the displacement in *dG*-norm converges with order $\ell +1$ (we remark that the expected order is $\ell$ in this case Theorem 3, [26, 27] and this rate is observed for the generalized-pressure). Moreover, for what concerns the error in $L^2$-norm, we observe $(\ell +1)+1$ convergence rate for the first refinements.

**Fig. 8:**
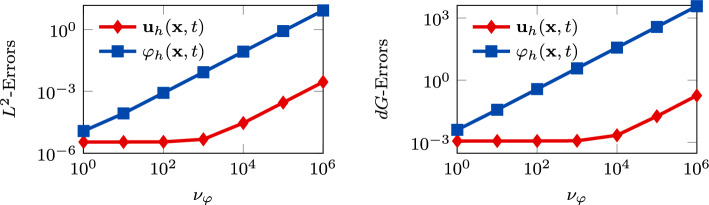
Superconvergence test: computed errors in $L_2$-norm (left) and *dG*-norm (right) versus $\nu \theta$ (*log*-*log* scale) using as polynomial degrees of approximation and number of elements: $= 2, N = 400$

In Figure 8, we observe the behavior of the errors with respect to increasing values of $\nu _{\varphi }$. We see that both the $L^2$- and *dG*-errors of the displacements are way lower than the errors of the generalized pressure (even for not too big values of $\nu _{\varphi }$). It is interesting to notice that, for the first tested values of $\nu _{\varphi }$, the displacement errors remain almost constant while the errors of the generalized pressure start growing.

### Robustness Tests

In this section, we address the problem of testing the scheme’s robustness with respect to some of the physical parameters appearing in (1). In particular, we are interested in testing the robustness of our method with respect to the coefficients for which the stability estimate in Theorem (2) is not robust: low values of the permeability ${\textbf{D}}$, low values of the second Lamé parameter $\mu$, and low values of the viscoelasticity retardation time $\delta _1$. In addition to that, we are interested in assessing the performance of the scheme with respect to high values of secondary consolidation $\delta _2 \lambda$, possibly considering low values of the storage coefficient $d_0$. Last, we test our method in the case of strong coupling between the momentum equation and the mass/energy one, by considering degenerating values of the first Lamé parameter $\lambda$. The values of the other model parameters are as in Table 2. The time discretization parameters are $T_f = 0.1$, $\Delta t = 5 \cdot 10^{-5}$. For the robustness analysis, a sequence of polygonal meshes as the one in Figure 1 is considered, and we consider polynomial degree $\ell = 3$.

**Table 4:** Robustness test vs conductivity: polynomial degree $\ell = 3$. The parameters are chosen as in Table 2 and the conductivity is set as ${\textbf{D}} = {10^{-6}{\textbf{I}}}$

1/*h*	$\Vert {\textbf{e}}^{u}\Vert _{L^2}$	$\text {roc}^{u}_{L^2}$	$\Vert {\textbf{e}}^{u}\Vert _{dG}$	$\text {roc}^{u}_{dG}$	$\Vert e^{p}\Vert _{L^2}$	$\text {roc}^{p}_{L^2}$	$\Vert e^{p}\Vert _{dG}$	$\text {roc}^{p}_{dG}$
5.53	${1.58 \cdot 10^{-5}}$	-	${4.63 \cdot 10^{-3}}$	-	${5.98 \cdot 10^{-4}}$	-	${1.77 \cdot 10^{-4}}$	-
7.62	${3.46 \cdot 10^{-6}}$	4.74	${1.58 \cdot 10^{-3}}$	3.36	${1.89 \cdot 10^{-4}}$	3.59	${7.22 \cdot 10^{-5}}$	2.80
10.53	${6.67 \cdot 10^{-7}}$	5.10	${4.91 \cdot 10^{-4}}$	3.61	${5.19 \cdot 10^{-5}}$	4.01	${2.95 \cdot 10^{5}}$	2.77
14.68	${1.84 \cdot 10^{-7}}$	3.88	${1.94 \cdot 10^{-4}}$	2.79	${2.00 \cdot 10^{-5}}$	2.87	${1.45 \cdot 10^{-5}}$	2.14
20.45	${4.42 \cdot 10^{-8}}$	4.30	${6.25 \cdot 10^{-5}}$	3.42	${6.58 \cdot 10^{-6}}$	3.35	${6.90 \cdot 10^{-6}}$	2.24

**Table 5:** Robustness test vs Lamé parameter $\mu$: polynomial degree $\ell = 3$. The parameters are chosen as in Table 2, the second Lamé parameter is set as $\mu = {10^{-6}}$

1/*h*	$\Vert {\textbf{e}}^{u}\Vert _{L^2}$	$\text {roc}^{u}_{L^2}$	$\Vert {\textbf{e}}^{u}\Vert _{dG}$	$\text {roc}^{u}_{dG}$	$\Vert e^{p}\Vert _{L^2}$	$\text {roc}^{p}_{L^2}$	$\Vert e^{p}\Vert _{dG}$	$\text {roc}^{p}_{dG}$
5.53	${3.02 \cdot 10^{-5}}$	-	${2.184 \cdot 10^{-3}}$	-	${1.95 \cdot 10^{-5}}$	-	${2.73 \cdot 10^{-3}}$	-
7.62	${1.13 \cdot 10^{-5}}$	3.06	${7.59 \cdot 10^{-4}}$	3.29	${5.54 \cdot 10^{-6}}$	3.92	${1.02 \cdot 10^{-3}}$	3.05
10.53	${3.59 \cdot 10^{-6}}$	3.55	${2.33 \cdot 10^{-4}}$	3.66	${1.27 \cdot 10^{-6}}$	4.56	${3.25 \cdot 10^{-4}}$	3.55
14.68	${9.33 \cdot 10^{-7}}$	4.05	${9.06 \cdot 10^{-5}}$	2.84	${3.61 \cdot 10^{-7}}$	3.78	${1.16 \cdot 10^{-4}}$	3.11
20.45	${3.53 \cdot 10^{-7}}$	2.93	${2.92 \cdot 10^{-5}}$	3.41	${8.31 \cdot 10^{-8}}$	4.43	${3.97 \cdot 10^{-5}}$	3.22

**Table 6:** Robustness test vs viscoelasticity retardation time $\delta _1$: polynomial degree $\ell = 3$. The parameters are chosen as in Table 2, the viscoelasticity retardation time is set as $\delta _1 = {10^{-6}}$

1/*h*	$\Vert {\textbf{e}}^{u}\Vert _{L^2}$	$\text {roc}^{u}_{L^2}$	$\Vert {\textbf{e}}^{u}\Vert _{dG}$	$\text {roc}^{u}_{dG}$	$\Vert e^{p}\Vert _{L^2}$	$\text {roc}^{p}_{L^2}$	$\Vert e^{p}\Vert _{dG}$	$\text {roc}^{p}_{dG}$
5.53	${2.11 \cdot 10^{-5}}$	-	${4.76 \cdot 10^{-3}}$	-	${2.30 \cdot 10^{-5}}$	-	${2.94 \cdot 10^{-3}}$	-
7.62	${4.43 \cdot 10^{-6}}$	4.86	${1.61 \cdot 10^{-3}}$	3.38	${5.68 \cdot 10^{-6}}$	4.35	${1.04 \cdot 10^{-3}}$	3.25
10.53	${8.96 \cdot 10^{-7}}$	4.95	${5.02 \cdot 10^{-4}}$	3.61	${1.32 \cdot 10^{-6}}$	4.52	${3.33 \cdot 10^{-4}}$	3.51
14.68	${2.27 \cdot 10^{-7}}$	4.13	${1.97 \cdot 10^{-4}}$	2.81	${3.69 \cdot 10^{-7}}$	3.82	${1.17 \cdot 10^{-4}}$	3.14
20.45	${5.55 \cdot 10^{-8}}$	4.24	${6.39 \cdot 10^{-5}}$	3.40	${8.50 \cdot 10^{-8}}$	4.43	${4.03 \cdot 10^{-5}}$	3.22

First, comparing the results for low values of ${\textbf{D}}$, $\mu$, and $\delta _1$ (cf. Table 4, Table 5, Table 6, respectively) with the error estimate presented in Theorem 3 and with classical error estimates of the PolyDG methods (see also Section 5.1), we observe that our scheme is robust with respect to these configuration. We observe that we lose $\ell +1$ accuracy in $L^2$-norm for the generalized pressure in the case ${\textbf{D}} \ll 1$ and for the displacement in the case $\mu \ll 1$, but we still observe convergence of order $\ell$ in both cases.

**Table 7:** Robustness test vs secondary consolidation: polynomial degree $\ell = 3$. The parameters are chosen as in Table 2 and the secondary consolidation coefficient is set as $\delta _2 \lambda = {10^{6}}$

1/*h*	$\Vert {\textbf{e}}^{u}\Vert _{L^2}$	$\text {roc}^{u}_{L^2}$	$\Vert {\textbf{e}}^{u}\Vert _{dG}$	$\text {roc}^{u}_{dG}$	$\Vert e^{p}\Vert _{L^2}$	$\text {roc}^{p}_{L^2}$	$\Vert e^{p}\Vert _{dG}$	$\text {roc}^{p}_{dG}$
5.53	${3.74 \cdot 10^{-4}}$	-	0.02	-	${2.00 \cdot 10^{-5}}$	-	${2.72 \cdot 10^{-3}}$	-
7.62	${1.31 \cdot 10^{-4}}$	3.28	${8.99 \cdot 10^{-3}}$	1.90	${5.63 \cdot 10^{-6}}$	3.94	${1.03 \cdot 10^{-3}}$	3.02
10.53	${3.36 \cdot 10^{-5}}$	4.21	${3.20 \cdot 10^{-3}}$	3.20	${1.28 \cdot 10^{-6}}$	4.58	${3.28 \cdot 10^{-4}}$	3.55
14.68	${6.66 \cdot 10^{-6}}$	4.86	${9.37 \cdot 10^{-4}}$	3.69	${3.69 \cdot 10^{-7}}$	3.75	${1.18 \cdot 10^{-4}}$	3.09
20.45	${2.11 \cdot 10^{-6}}$	3.47	${3.98 \cdot 10^{-4}}$	2.579	${8.46 \cdot 10^{-8}}$	4.44	${4.04 \cdot 10^{-5}}$	3.22

**Table 8:** Robustness test vs secondary consolidation: polynomial degree $\ell = 3$. The parameters are chosen as in Table 2, the secondary consolidation coefficient is set as $\delta _2 \lambda = {{10^{6}}}$, and the storage coefficient is set as $d_0 = 10^{-6}$

1/*h*	$\Vert {\textbf{e}}^{u}\Vert _{L^2}$	$\text {roc}^{u}_{L^2}$	$\Vert {\textbf{e}}^{u}\Vert _{dG}$	$\text {roc}^{u}_{dG}$	$\Vert e^{p}\Vert _{L^2}$	$\text {roc}^{p}_{L^2}$	$\Vert e^{p}\Vert _{dG}$	$\text {roc}^{p}_{dG}$
5.53	${3.74 \cdot 10^{-4}}$	-	0.02	-	0.01	-	1.06	-
7.62	${1.31 \cdot 10^{-4}}$	3.28	${8.99 \cdot 10^{-3}}$	1.90	${3.00 \cdot 10^{-3}}$	4.65	0.33	3.59
10.53	${3.36 \cdot 10^{-5}}$	4.21	${3.20 \cdot 10^{-3}}$	3.20	${4.62 \cdot 10^{-4}}$	5.92	0.07	4.71
14.68	${6.68 \cdot 10^{-6}}$	4.85	${9.36 \cdot 10^{-4}}$	3.69	${1.07 \cdot 10^{-4}}$	4.41	0.02	3.59
20.45	${2.20 \cdot 10^{-6}}$	3.35	${3.98 \cdot 10^{-4}}$	2.58	${6.10 \cdot 10^{-5}}$	1.68	${5.00 \cdot 10^{-3}}$	4.45

For what concerns the *dG*-error analysis, in the case of small Lamé parameter $\mu$ and small viscoelasticity retardation time $\delta _1$ the errors decay as expected, while in the case of small conductivity ${\textbf{D}}$, we observe a decrease of the *dG*-errors of the pressure when refining the mesh. However, we observe that their values are affected by the fact that the conductivity value enters the norm’s definition. Then, we observe the performance of the method for high values of the secondary consolidation coefficient $\delta _2 \lambda$, considering both the cases in which we have not-degenerating (cf. Table 7) and degenerating storage coefficient $d_0$ (cf. Table 8). The method is robust in both regimes; however – as expected – it behaves slightly worse when the storage coefficient is $\ll 1$. Finally, we observe that our method is robust also in the case of strong coupling between the two equations of the model problem, cf. Table 9.

**Table 9:** Robustness test vs coupling strength: polynomial degree $\ell = 3$. The parameters are chosen as in Table 2, the first LamÃ¨ parameter has been set as $\lambda = {10^{-6}}$

1/*h*	$\Vert {\textbf{e}}^{u}\Vert _{L^2}$	$\text {roc}^{u}_{L^2}$	$\Vert {\textbf{e}}^{u}\Vert _{dG}$	$\text {roc}^{u}_{dG}$	$\Vert e^{p}\Vert _{L^2}$	$\text {roc}^{p}_{L^2}$	$\Vert e^{p}\Vert _{dG}$	$\text {roc}^{p}_{dG}$
5.53	${1.56 \cdot 10^{-5}}$	-	${4.14 \cdot 10^{-3}}$	-	${4.97 \cdot 10^{-5}}$	-	${4.57 \cdot 10^{-3}}$	-
7.62	${3.38 \cdot 10^{-6}}$	4.76	${1.40 \cdot 10^{-3}}$	3.38	${1.03 \cdot 10^{-5}}$	4.92	${1.49 \cdot 10^{-3}}$	3.50
10.53	${6.46 \cdot 10^{-7}}$	5.12	${4.38 \cdot 10^{-4}}$	3.60	${2.10 \cdot 10^{-6}}$	4.91	${4.33 \cdot 10^{-4}}$	3.82
14.68	${1.79 \cdot 10^{-7}}$	3.86	${1.73 \cdot 10^{-4}}$	2.79	${5.74 \cdot 10^{-7}}$	3.90	${1.57 \cdot 10^{-4}}$	3.04
20.45	${4.30 \cdot 10^{-8}}$	4.30	${5.58 \cdot 10^{-5}}$	3.41	${1.39 \cdot 10^{-7}}$	4.28	${5.35 \cdot 10^{-5}}$	3.26

#### Remark 5

In Theorem 2, we observe that the stability estimate is not robust with respect to ${\textbf{D}}$ and the product between $\mu$ and $\delta _1$. However, in Table 4, Table 5, and Table 6, we have shown that the proposed method is robust with respect to low values of these parameters.

### Wave Propagation in Thermo-elastic Media

This section considers a wave propagation problem in thermoelastic media inspired by [28]. The aim of this simulation is to prove that the proposed scheme can reproduce known results present in the literature for the thermoelastic framework and can give *physically-sound* results.

*Vertical source term.* We consider a domain $\Omega = (0\,\textrm{m}, 2310\, \textrm{m})^2$ with the thermoelastic properties reported in Table 10 [28].

**Table 10:** Wave propagation in thermoelastic media: homogeneous medium properties

**Coefficient**	**Value**		**Coefficient**	**Value**
$\rho [\hbox {kg\,m}^{3}]$	2650		$\beta [\hbox {Pa\,K}^{-1}]$	79200
$\mu$ [Pa]	$6 \cdot 10^{9}$		$\lambda [\hbox {Pa}]$	$4 \cdot 10^{9}$
$a_0 [\hbox {Pa}\,K^{-2}]$	117		$\varvec{\Theta } [\hbox {m}^{2}\,\hbox {Pa}\,\hbox {K}^{-2}\hbox {s}^{-1}]$	10.5 ${\textbf{I}}$
$\tau _1 [\hbox {s}]$	${1.49 \cdot 10^{-8}}$		$\tau _2 [\hbox {s}]$	${1.49 \cdot 10^{-8}}$
$\delta _1 [\hbox {s}]$	0		$\delta _2 [\hbox {s}]$	0

In the first test case, we consider a vertical source term ${\textbf{f}}$ in (1a). The source is set in the computational domain’s center and multiplied by a time-history function *h*(*t*). In our case, the time evolution is given by [28] $h(t) = A_0 \cos \left[ 2 \pi (t - t_0) f_0 \right] \exp \left[ -2 (t - t_0)^2 f_0^2 \right]$, where $A_0 = 10^{4}$ m is the amplitude, $f_0 = 5$ Hz is the peak-frequency, and $t_0 = 3/(2 f_0) = 0.3$ s is the time-shift. We adopt a polygonal mesh with mesh size $h \sim 76$ m (3500 elements) and polynomial degree $\ell = 4$. As a time-stepping scheme we employ the Newmark-$\beta$ scheme, with $\Delta t = {5 \cdot 10^{-4}}$ s and $T_f = 0.5$ s. Finally, we complete our problem with homogeneous Dirichlet boundary conditions and null initial conditions. In the following, we denote by ${\textbf{v}}_h$ the solid velocity (i.e. $\dot{ {\textbf{u}}}_h$) and by $v_{h,y}$ its vertical component.

**Fig. 9:**
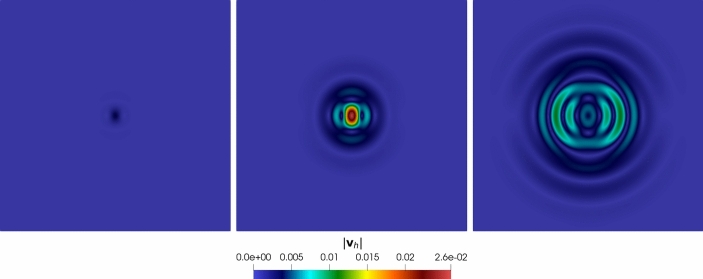
Wave propagation in thermoelastic media with vertical source term: computed velocity field |**v***h* | at the time instants $t = 0.1$s (left), $t = 0.3$s (center), $t = 0.5$s (right)

**Fig. 10:**
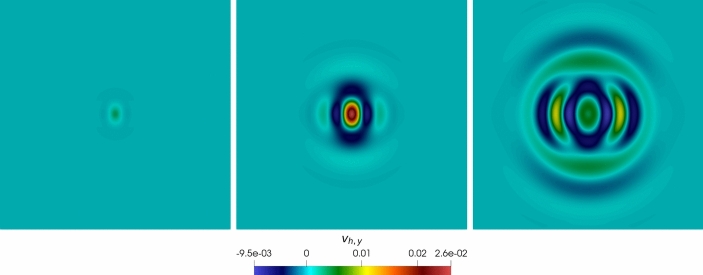
Wave propagation in thermoelastic media with vertical source term: computed vertical component of the velocity field $\textit{vh},y$ at the time instants $t = 0.1$s (left), $t = 0.3$s (center), $t = 0.5$s (right)

From the results of Figure 8 we observe a symmetric wavefront that detaches from the center of the domain symmetric to the *x*- and *y*-axes. This behavior is correct due to the form of the forcing term we are imposing. Looking at the three snapshots, we can observe the presence of the elastic *E*-wave captured by our scheme and the *S*-wave. The presence of these two waves is more evident by looking at Figure 9, where the computed vertical velocity field is reported. Indeed, it is possible to observe the propagation of the *E* wave, traveling faster and along the *y*-direction, and the propagation of the *S*-waves that are slower and propagate along the *x*-direction. Last, in Figure 10, we observe the propagation of the diffusive *T*-wave, that is originated by a vertical source term in the momentum conservation equation. A thermal source term in the energy conservation equation is not considered here. In conclusion, considering the different central peak frequencies $f_0$, we can see a good agreement between our results and those presented in [29].

*Shear source term.* In the second test case, we model the forcing term of the momentum equation ${\textbf{f}}$ as a shear source. The forcing term is modeled as ${\textbf{f}} = - {\textbf{M}} \, \nabla \hspace{-1.38885pt}\cdot \hspace{-1.38885pt}{\delta }({\textbf{x}} - {\textbf{x}}_s) \, h(t)$ [49], where ${\textbf{M}}$ is a moment tensor, ${\textbf{x}}_s$ is the point-source location, $\delta ({\textbf{x}} - {\textbf{x}}_s)$ is the Kronecker delta located in ${\textbf{x}}_s$, and *h*(*t*) is the aforementioned time-history function. We consider ${\textbf{M}}$ a tensor with zero components on the diagonal and not zero components off-diagonal; this choice induces the presence of shear waves and generally has a strong connection with the wave patterns we observe. A momentum source of this shape is often used in the context of earthquakes. The properties of the medium and the discretization parameters are chosen as in the vertical source term test.

**Fig. 11:**
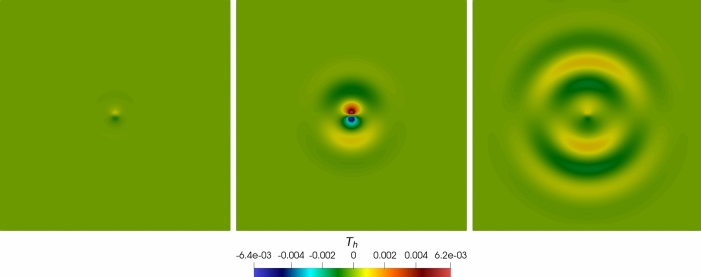
Wave propagation in thermoelastic media with vertical source term: computed temperature field Th at the time instants $t = 0.1$s (left), $t = 0.3$s (center), $t = 0.5$s (right)

**Fig. 12:**
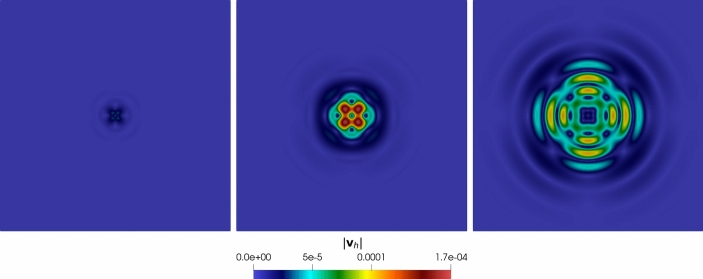
Wave propagation in thermoelastic media with shear source term: computed velocity field $|{\textbf {v}}h |$ at the time instants $t = 0.1$s (left), $t = 0.3$s (center), $t = 0.5$s (right)

**Fig. 13:**
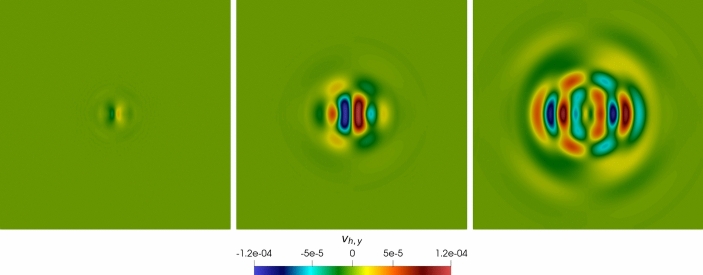
Wave propagation in thermoelastic media with shear source term: computed vertical component of the velocity field *vh*, *y* at the time instants $t = 0.1$s (left), $t = 0.3$s (center), $t = 0.5$s (right)

From the results of Figure 11 we notice a symmetric wavefront that detaches from the center of the domain. The velocity field has a symmetric pattern with respect to the diagonals of the domain (due to the choice of the forcing term). Looking at the three snapshots, we observe the propagation of the elastic wave. From the results in Figure 12, we notice the presence of shear waves and the anti-symmetric pattern of the wave fronts with respect to the *y*-axis. In Figure 13, we can see the presence of the diffusive thermal *T*-wave, which has a symmetric pattern with respect to the diagonals of the domain and antisymmetric with respect to the *x*- and *y*-axis. The results we obtain for this test are coherent with [28] (thermoelasticity) and [19, 29] (thermo-poroelasticity).

*Shear source term in a heterogeneous media.* In this last thermoelastic test case, we model the thermoelastic wave propagation in a heterogeneous media. We split the domain $\Omega = (-1155\,\textrm{m}, 1155\,\textrm{m}) \times (0\,\textrm{m}, 2310\,\textrm{m})$ into two vertical layers. The left layer is characterized by the same thermoelastic properties of the test case *Shear source term*, while in the right layer, we consider the following: cf. Table 11 (the parameters that are not listed there are taken as in Table 10). This final test case aims to investigate how the heterogeneity of the media can affect the wave propagation phenomena. The forcing terms, and discretizations parameters are the same of *Shear source term*-test case.

**Table 11:** Wave propagation in thermoelastic media: heterogeneous media properties (right layer). The thermoelastic properties of the left layer are reported in Table 10

**Coefficient**	**Value**		**Coefficient**	**Value**
$\mu [\hbox {Pa}]$	$10^{9}$		$\lambda [\hbox {Pa}]$	${6.5\, \cdot 10^{8}}$
$a_0 [\hbox {Pa}\,\hbox {K}^{-2}]$	20		$\varvec{\Theta } [\hbox {m}^{2}\,\hbox {Pa}\,\hbox {K}^{-2}\hbox {s}^{-1}]$	5 ${\textbf{I}}$
$\tau _1 [\hbox {s}]$	$2.5\,\cdot \,10^{-7}$		$\tau _2 [\hbox {s}]$	$2.5\,\cdot \,10^{-7}$

**Fig. 14:**
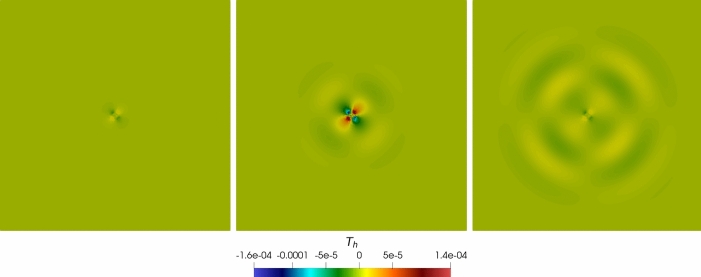
Wave propagation in thermoelastic media with shear source term: computed temperature field *Th* at the time instants $t = 0.1$s (left), $t = 0.3$s (center), $t = 0.5$s (right)

**Fig. 15:**
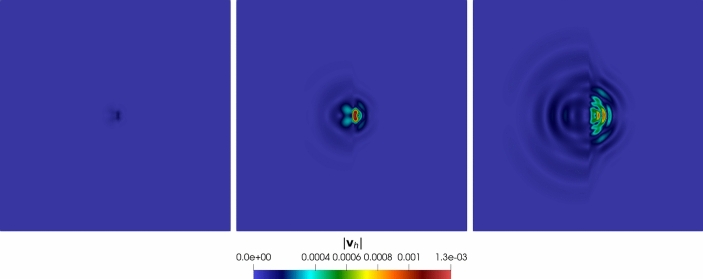
Wave propagation in thermoelastic media with shear source term: computed velocity field $|{\textbf {v}}h |$ at the time instants $t = 0.1$s (left), $t = 0.3$s (center), $t = 0.5$s (right)

**Fig. 16:**
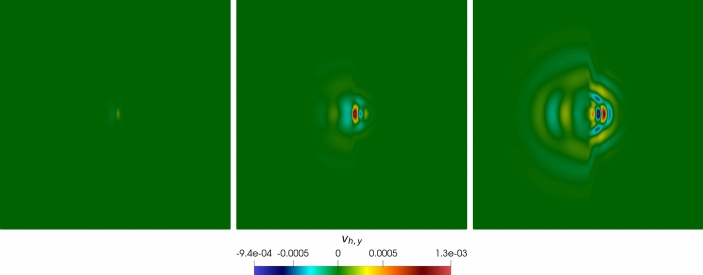
Wave propagation in thermoelastic media with shear source term: computed vertical component of the velocity field *vh*, *y* at the time instants $t = 0.1$s (left), $t = 0.3$s (center), $t = 0.5$s (right)

In terms of the velocity field – displayed in Figure 14 and Figure 15 – the main difference with respect to the homogeneous case is the presence of the head waves that connect the wavefronts of the two sub-domains. The head waves are particularly evident by looking at the vertical component of the velocity field (cf. last frame of Figure 15). For what concerns the temperature field $T_h$, whose behavior is shown in Figure 16, we observe how the increasing value in the thermal conductivity affects the profile of the *T*-wave and, even in this case, we can observe the presence of wavelets that connect the wavefronts in the two layers. In general, by looking at the results for all three fields, we can observe that the heterogeneities in the media properties are well resolved by our method and that all the symmetric and anti-symmetric patterns observed in the *Shear source term*-test case are preserved. However, the wave propagation in the two sub-layers is non-identical due to the different physical properties of the two media. We can observe a qualitatively good agreement with the results presented in [28] for the thermoelastic wave propagation and in [19, 29] for the thermo-poroelastic wave propagation.

### Fluid Flow in Heterogeneous Poro-viscoelastic Media

In this section, we consider the injection of a fluid in a heterogeneous poro-viscoelastic medium inspired by the SPE10 benchmark [32]. This simulation aims to analyze the impact of the different possible modeling choices on the fluid flows in geophysical applications. We increase the initial permeability values by four orders to highlight the impact in high-permeable channels.

**Fig. 17:**
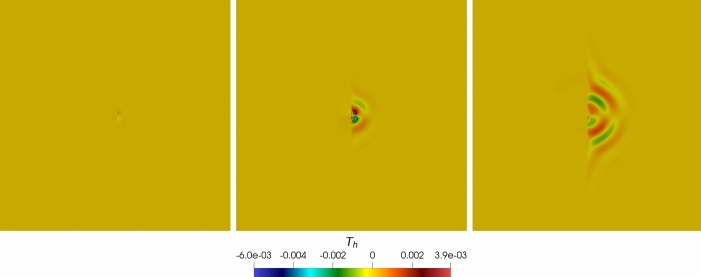
Wave propagation in thermoelastic media with shear source term: computed temperature field Th at the time instants $t = 0.1$s (left), $t = 0.3$s (center), $t = 0.5$s (right)

We consider as domain a horizontal slice (number 35) of the SPE10 benchmark $\Omega = (0\,\textrm{m}, 366\,\textrm{m})\times (0\,\textrm{m}, 671\,\textrm{m})$. The permeability values associated with the simulations can be observed in Figure 17 (left). It can be observed that there is a channel of higher permeability that we expect to transport most of the fluid flow inside the domain. The simulation is associated with an injection of fluid in two sources with reabsorption in the middle of the domain. To reproduce this phenomenon, we set a forcing term:

$$\begin{aligned} g({\textbf{x}},t) = \dfrac{\tanh (5 t)}{10} \left( e^{-\frac{(x-190)^2+(y-550)^2}{500}}+e^{-\frac{(x-130)^2+(y-120)^2}{500}}-e^{-\frac{(x-175)^2+(y-360)^2}{500}}\right) . \end{aligned}$$

With this choice, the injection and absorption increase in time until $t=0.5 \textrm{s}$ when they reach a plateau. Concerning initial conditions, we set pressure, displacement, and velocity equal to 0. Moreover, we set homogeneous Dirichlet boundary conditions for the displacement and homogeneous Neumann boundary conditions for the pressure. Concerning the space discretization, we use the cartesian mesh of 13200 elements provided by the SPE10 benchmark ($h=6.81\,\textrm{m}$). This choice lets us maintain the initial benchmark’s tensor ${\textbf{D}}$ refinement level. We use the polynomial degree $\ell =2$ and a timestep $\Delta t=4 \cdot 10^{-4}$.

**Table 12:** Fluid flow in heterogeneous poro-viscoelastic medium: physical parameters in the three simulation settings.

**Parameter**		**Model PVE**	**Model P**	**Model D**	**Reference**
$\mu$	$[\hbox {Pa}]$	$10^9$	-	-	[19]
$\lambda$	$[\hbox {Pa}]$	$4\times 10^8$	-	-	[19]
$\delta _1$	$[\hbox {s}]$	$8\times 10^{-5}$	-	-	[53]
$\delta _2$	$[\hbox {s}]$	$8\times 10^{-5}$	-	-	[53]
$\gamma$	$[-]$	1	0	0	[19]
$d_0$	$[\hbox {Pa}^{-1}]$	$10^{-9}$	$10^{-9}$	$10^{-9}$	[19]
$\tau _1$	$[\hbox {s}]$	$\rho _f \phi ^{-1} {\textbf{D}}$	$\rho _f \phi ^{-1} {\textbf{D}}$	0	[52]
$\tau _2$	$[\hbox {s}]$	$\rho _f \phi ^{-1} {\textbf{D}}$	$\rho _f \phi ^{-1} {\textbf{D}}$	0	[52]
$\rho _f$	$[\hbox {kg\,m}^{-3}]$	$1.025\times 10^3$	$1.025\times 10^3$	$1.025\times 10^3$	[43]
$\phi$	$[-]$	0.1	0.1	0.1	[52]

Starting from this general setting, which is common to all the simulations in this subsection, we construct three different models. In particular, we compare the proposed poro-viscoelastic model (PVE) with two porous media models: the first comprehensive of the acceleration term of the Darcy law (model P) and the second with a classical static Darcy law (model D). All these models can be obtained in our general framework by taking different values of the physical parameters. The values used in this simulation with the specific references are reported in Table 12.

**Fig. 18:**
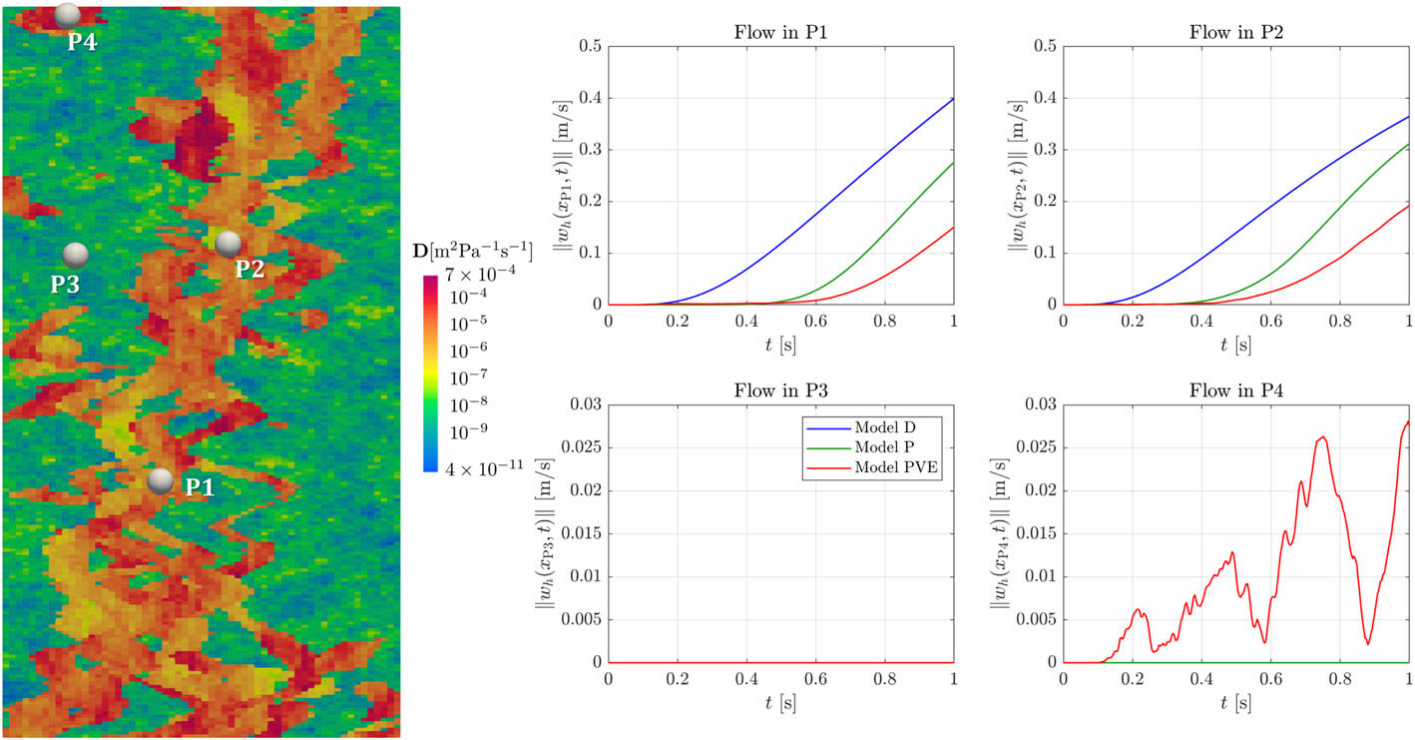
Fluid flow in heterogeneous porous-viscoelastic medium: computed filtration velocity field ${\textbf {w}}h$ measured in specific points of the domain with the three different models

In Figure 17, we report the result of the three models regarding the magnitude of the fluid filtration $\Vert {\textbf{w}}_h\Vert = \Vert {\textbf{D}} \nabla p_h\Vert$. In particular, we report the values measured at four specific points inside the domain. Concerning points P1$=(146.30\,\textrm{m},236.22\,\textrm{m})$ and P2$=(207.26\,\textrm{m},452.63\,\textrm{m})$, we can notice that the magnitude increases faster in model D than in the others. This is coherent with the modeling of the Darcy law, which does not consider fluid acceleration. This choice penalizes the fluid flow’s continuity, reducing the previous timesteps’ effect on the flow. Observing the model PVE, we highlight that the viscoelasticity reduces the magnitude of the flow in points P1 and P2, creating an additional delay in the fluid flow development. Concerning point P3$=(67.05\,\textrm{m},441.69\,\textrm{m})$, where the permeability is lower than in the other recording points, the fluid flow is approximately null in all the models (in the PVE model, the values are $\simeq 10^{-6} \mathrm {m/s}$). Finally, in P4$=(60.96\,\textrm{m},661.42\,\textrm{m})$, the elastic deformation of the soil induces a fluid flow in the model PVE, although the area is quite far from the channel in which the fluid injection is modeled.

In Figure 18, we report the complete fluid filtration field at three-time instants ($t=0.2,0.5,1.0\,\textrm{s}$) and for the three different models. In particular, the glyphs are not reported wherever the flow is null. We can observe that the gap between models PVE and P against model D reduces during the simulation after a significant distance at the first times ($t=0.2\,\textrm{s}$). In all the cases, the fluid flow is located along the high-permeability channel inside the domain. However, a significant difference is fluid flow induction inside non-connected high-permeability regions in model PVE, which increases over time.

**Fig. 19:**
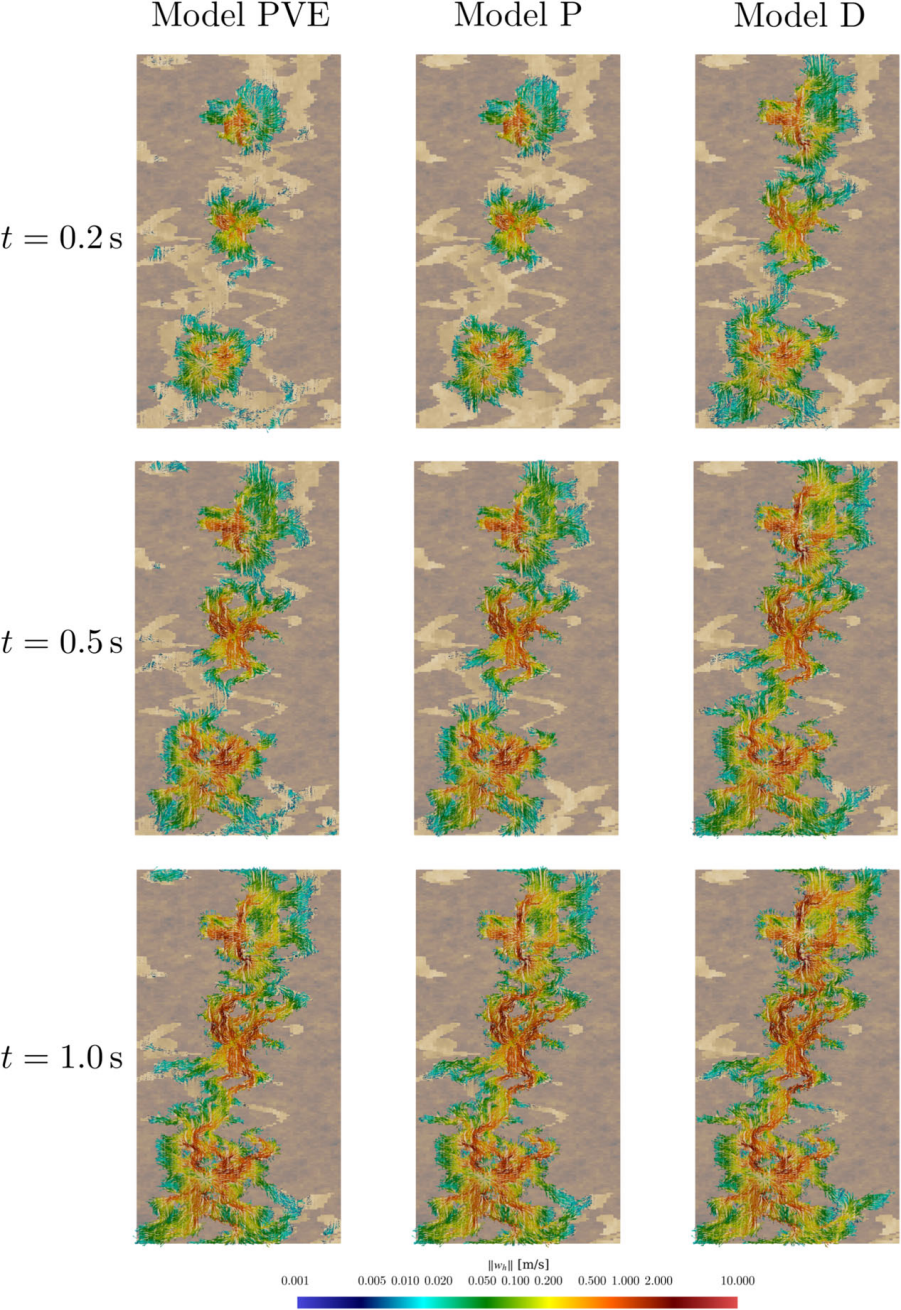
Fluid flow in heterogeneous porous-viscoelastic medium: computed filtration velocity field wh at the time instants $t = 0.2$ s (top), $t = 0.5$ s (middle), and $t = 1.0$ s (bottom). Glyphs are not present wherever the flow is absent. Three different models are reported: model PVE (left), model P (middle), and model D (right)

To quantify the differences between the modeling choices, we construct a relative measure of filtration distance. Indeed, considering the models $*$ and $'$, we introduce for each mesh element $\kappa$:

$$\begin{aligned} \Vert {\textbf{w}}_h^*-{\textbf{w}}_h'\Vert _r = \dfrac{\Vert {\textbf{w}}_h^*-{\textbf{w}}_h'\Vert _K}{\Vert {\textbf{w}}_h^\textrm{D}\Vert }, \end{aligned}$$

where the choice of rescaling with $\Vert {\textbf{w}}_h^\textrm{D}\Vert$ is guided by the fact this is the *state-of-the-art* model in literature. In Figure 19 (on the right), we report the relative differences in flow magnitude between the three models. As we can observe at time $t=0.5\,\textrm{s}$, in points with high permeability values, the Darcy model overestimates the flow by more than $100\%$. In Figure 19 (on the left), we report the mean in space (with confidence bounds computed with the standard deviation) for each time. We can observe that the difference between D and P, as well as between D and PVE, decreases in time. This is coherent with the additional inertia introduced by the terms $\tau _1$ and $\tau _2$. On the contrary, the difference between P and PVE is smaller than the others and almost constant in time. Indeed, after an initial time, when the models provide almost the same solution, the difference is highly guided by the capability of PVE to catch the flow in high permeability regions detached from the central channel. Finally, we underline that neglecting components of the mathematical model in choosing the Darcy law can sensibly affect the final solution.

## Conclusions

In this work, we have presented a PolyDG formulation for the thermo/poro-viscoelasticity problem. The model derivation remarks on which terms are more or less significant depending on the physical parameters of our test cases and, consequently, on the reference application. The stability estimate in the semi-discrete framework highlights the mild requirements on the physical parameters of the model problem. Moreover, it is general with respect to the choice of a fully-inertial or quasi-static model. For the semi-discrete framework, an a-priori error estimate is provided. Numerical simulations are performed to test the convergence and robustness properties of the proposed method. The results also show that the method provides a good approximation when considering limit cases in the ranges of physical parameters and presents some partial-superconvergence results. Finally, some benchmark verifications and physically sound test cases are presented, showing that the PolyDG discretization scheme can be appealing for real problem simulations with appropriate parameters. In particular, we used the SPE10 benchmark to highlight the impact of modeling choice on the fluid flow in heterogeneous media in geophysics.

**Fig. 20:**
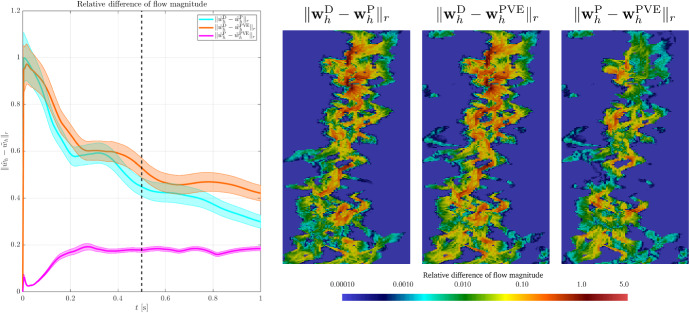
Relative difference of flow magnitude: mean values and confidence bounds for each time (left) and detailed differences at time 0.5 s.

Further developments of the present work are possible. In particular, we mention the extension to other non-linear models for visco-elasticity. Secondly, to reduce the large computational cost required to deal with the fully coupled problem, two possible approaches are designing effective preconditioning techniques for the resulting system and developing effective splitting schemes. Given the improvements in the linear system resolution strategy, extending the implementation to the three-dimensional case is a further possible improvement. For this, generating and managing the computational mesh is also a point of development and interest. Finally, it would be interesting to analyze the complete thermo-poro-viscoelastic model, which has several applications. From a mathematical point of view, the coupling between the fluid and the temperature fields would provide interesting additional challenges, such as the presence of the nonlinear temperature convective term.

## Data Availability

Enquiries about data availability should be directed to the authors.

## Derivation of Newmark-$\beta$-$\theta$ Method

In this appendix, we show the computations to derive the Newmark-$\beta$-$\theta$ method for discretizing the equations with $\tau _1=0$. We use the Newmark-$\beta$ method for the first equation of (12). To this aim, we introduce the velocity vector ${\textbf{Z}} = \dot{{\textbf{U}}}$ and the acceleration vector ${\textbf{A}} = \ddot{{\textbf{U}}}$. Then, at each time-step $t^n$, we solve the following set of equations:

15 $$\begin{aligned} \left\{ \begin{aligned}&\begin{aligned} \bigg ( \frac{1}{\beta _N \Delta t^2} {\textbf{M}}_u&+ {\textbf{A}}_e + {\textbf{A}}_{\text {div}} \bigg ) {\textbf{U}}^{n+1} + \left( {\textbf{A}}_{e,\delta _1} + {\textbf{A}}_{\text {div},\delta _2} \right) {\textbf{Z}}^{n+1} - {\textbf{C}}^T \varvec{\Phi }^{n+1}\\&= {\textbf{F}}^{n+1} + \frac{1}{\beta _N \Delta t^2} {\textbf{M}}_u {\textbf{U}}^n \\&+ \frac{1}{\beta _N \Delta t} {\textbf{M}}_u {\textbf{Z}}^n + \frac{1 - 2\beta _N}{2\beta _N} {\textbf{M}}_u {\textbf{A}}^n, \end{aligned}\\&{\textbf{Z}}^{n+1} = {\textbf{Z}}^{n} + \Delta t \left( \gamma _N {\textbf{A}}^{n+1} + (1-\gamma _N) {\textbf{A}}^n \right) , \\&{\textbf{A}}^{n+1} = \frac{1}{\beta _N \Delta t^2}\left( {\textbf{U}}^{n+1} - {\textbf{U}}^{n}\right) - \frac{1}{\beta _N \Delta t} {\textbf{Z}}^{n} + \frac{2\beta _N - 1}{2\beta _N} {\textbf{A}}^n. \end{aligned} \right. \end{aligned}$$

In the first equation of (15) we can plug the expressions for ${\textbf{Z}}^{n+1}$, ${\textbf{A}}^{n+1}$ to get:

$$\begin{aligned} \begin{aligned}&\bigg ( \frac{1}{\beta _N \Delta t^2} {\textbf{M}}_u + {\textbf{A}}_u + \frac{\gamma _N}{\beta _N \Delta t} {\textbf{A}}_{u, \delta } \bigg ) {\textbf{U}}^{n+1} - {\textbf{C}}^T \varvec{\Phi }^{n+1} \\&\quad = {\textbf{F}}^{n+1} + \left( \frac{1}{\beta _N \Delta t^2} {\textbf{M}}_u + \frac{\gamma _N}{\beta _N \Delta t} {\textbf{A}}_{u, \delta } \right) {\textbf{U}}^{n} \\&+ \bigg ( \frac{1}{\beta _N \Delta t} {\textbf{M}}_u - \frac{\beta _N - \gamma _N}{\beta _N} {\textbf{A}}_{u, \delta } \bigg ) {\textbf{Z}}^n + \left( \frac{1 - 2\beta _N}{2\beta _N} {\textbf{M}}_u - \frac{\Delta t (2\beta _N-\gamma _N)}{2 \beta _N} {\textbf{A}}_{u, \delta } \right) {\textbf{A}}^n, \\ \end{aligned} \end{aligned}$$

where, for the sake of clarity, we have introduced the notation ${\textbf{A}}_{u} = {\textbf{A}}_{e} + {\textbf{A}}_{\text {div}}$, ${\textbf{A}}_{u, \delta } = {\textbf{A}}_{e,\delta _1} + {\textbf{A}}_{\text {div},\delta _2}$. Then, we couple (15) with a $\theta$-method for the pressure equation. To obtain the formulation, we plug into the second equation in (12) the definition of the velocity ${\textbf{Z}}$ and of the acceleration ${\textbf{A}}$ at time-continuous level. Thus, we get the following expression:

$$\begin{aligned} {\textbf{M}}_{\varphi } \, \dot{\varvec{\Phi }}(t) + {\textbf{C}}_{\tau _2} \, {\textbf{A}}(t) + {\textbf{C}} \, {\textbf{Z}}(t) + {\textbf{A}}_{\varphi } \varvec{\Phi }(t) = {\textbf{G}}(t), \quad t \in (0,T_f]. \end{aligned}$$

Using this form of the pressure equation, the discretized equation reads:

$$\begin{aligned} \begin{aligned}&\left( \frac{1}{\Delta t}{\textbf{M}}_{\varphi } + \theta {\textbf{A}}_{\varphi } \right) \varvec{\Phi }^{n+1} + \theta \left( {\textbf{C}}_{\tau _2} \, {\textbf{A}}^{n+1} + {\textbf{C}} \, {\textbf{Z}}^{n+1} \right) = \left( \frac{1}{\Delta t}{\textbf{M}}_{\varphi } - (1-\theta ) {\textbf{A}}_{\varphi } \right) \varvec{\Phi }^{n} + \theta {\textbf{G}}^{n+1} \\&+ (1-\theta ) \left( {\textbf{G}}^{n} - {\textbf{C}}_{\tau _2} \, {\textbf{A}}^{n} - {\textbf{C}} \, {\textbf{Z}}^{n} \right) \end{aligned} \end{aligned}$$

and by using the expressions for ${\textbf{Z}}^{n+1}, {\textbf{A}}^{n+1}$ in (15) we obtain:

$$\begin{aligned} \begin{aligned}&\left( \frac{1}{\Delta t}{\textbf{M}}_{\varphi } + \theta {\textbf{A}}_{\varphi } \right) \varvec{\Phi }^{n+1} + {\textbf{C}}_u \, {\textbf{U}}^{n+1} = \theta \, {\textbf{G}}^{n+1} + {\tilde{\theta }} \, {\textbf{G}}^n + \left( \frac{1}{\Delta t}{\textbf{M}}_{\varphi } - {\tilde{\theta }} {\textbf{A}}_{\varphi } \right) \varvec{\Phi }^{n} \\&+ {\textbf{C}}_u \, {\textbf{U}}^{n} + {\textbf{C}}_z \, {\textbf{Z}}^{n} + {\textbf{C}}_a \, {\textbf{A}}^{n}, \end{aligned} \end{aligned}$$

where:

$$\begin{aligned} \begin{aligned}&{\textbf{C}}_u = \frac{\theta }{\beta _N \Delta t^2}\left( {\textbf{C}}_{\tau _2} + \Delta t \gamma {\textbf{C}} \right) , \\&{\textbf{C}}_z = \left( \frac{\theta }{\beta _N \Delta t} {\textbf{C}}_{\tau _2} + \frac{\theta \gamma _N - \beta _N}{\beta _N}{\textbf{C}} \right) , \\&{\textbf{C}}_a = \left( \frac{\theta - 2\beta _N}{2\beta _N} {\textbf{C}}_{\tau _2} + \frac{\theta \Delta t(\gamma - 2\beta _N)}{2\beta _N} {\textbf{C}} \right) , \end{aligned} \end{aligned}$$

and ${\tilde{\theta }} = 1 - \theta$. Thus, the final algebraic discretized formulation reads:

$$\begin{aligned} \left\{ \begin{aligned}&\begin{aligned} \bigg ( \frac{1}{\beta _N \Delta t^2} {\textbf{M}}_u + {\textbf{A}}_u + \frac{\gamma _N}{\beta _N \Delta t} {\textbf{A}}_{u, \delta } \bigg ) {\textbf{U}}^{n+1} - {\textbf{C}}^T \varvec{\Phi }^{n+1} = {\textbf{F}}^{n+1} + \left( \frac{1}{\beta _N \Delta t^2} {\textbf{M}}_u + \frac{\gamma _N}{\beta _N \Delta t} {\textbf{A}}_{u, \delta } \right) {\textbf{U}}^{n} \\ + \bigg ( \frac{1}{\beta _N \Delta t} {\textbf{M}}_u - \frac{\beta _N - \gamma _N}{\beta _N} {\textbf{A}}_{u, \delta } \bigg ) {\textbf{Z}}^n + \left( \frac{1 - 2\beta _N}{2\beta _N} {\textbf{M}}_u - \frac{\Delta t (2\beta _N-\gamma _N)}{2 \beta _N} {\textbf{A}}_{u, \delta } \right) {\textbf{A}}^n, \\ \end{aligned} \\&\begin{aligned} \left( \frac{1}{\Delta t}{\textbf{M}}_{\varphi } + \theta {\textbf{A}}_{\varphi } \right) \varvec{\Phi }^{n+1} + {\textbf{C}}_u \, {\textbf{U}}^{n+1} = \theta \, {\textbf{G}}^{n+1} + {\tilde{\theta }} \, {\textbf{G}}^n + \left( \frac{1}{\Delta t}{\textbf{M}}_{\varphi } - {\tilde{\theta }} {\textbf{A}}_{\varphi } \right) \varvec{\Phi }^{n} \\ + {\textbf{C}}_u \, {\textbf{U}}^{n} + {\textbf{C}}_z \, {\textbf{Z}}^n + {\textbf{C}}_a \, {\textbf{A}}^n, \end{aligned} \\&{\textbf{Z}}^{n+1} = {\textbf{Z}}^{n} + \Delta t \left( \gamma _N {\textbf{A}}^{n+1} + (1-\gamma _N) {\textbf{A}}^n \right) ,\\&{\textbf{A}}^{n+1} = \frac{1}{\beta _N \Delta t^2}\left( {\textbf{U}}^{n+1} - {\textbf{U}}^{n}\right) - \frac{1}{\beta _N \Delta t} {\textbf{Z}}^{n} + \frac{2\beta _N - 1}{2\beta _N} {\textbf{A}}^n, \end{aligned} \right. \end{aligned}$$

## Proof of Convergence Estimate (Theorem 3)

In this appendix, we report the proof of Theorem 3. To start the proof of convergence, we define the following quantities

$$\begin{aligned} \begin{aligned}&{\textbf{e}}_\textrm{I}^{{\textbf{u}}} = {\textbf{u}}-{\textbf{u}}_\textrm{I}, \quad e_\textrm{I}^\varphi = \varphi -\varphi _\textrm{I}, \quad {\textbf{e}}_\textrm{I}^{\dot{{\textbf{u}}}} = \dot{{\textbf{u}}}-\dot{{\textbf{u}}}_\textrm{I}, \quad {\textbf{e}}_\textrm{I}^{{\textbf{W}}} = {\textbf{W}} - {\textbf{W}}_\textrm{I}, \quad {\textbf{e}}_\textrm{I}^{\Psi } = \Psi -\Psi _\textrm{I}, \\&{\textbf{e}}_h^u = {\textbf{u}}_\textrm{I}-{\textbf{u}}_{h}, \quad e_h^\varphi = \varphi _\textrm{I}-\varphi _\textrm{h}, \quad {\textbf{e}}_h^{\dot{{\textbf{u}}}} = \dot{{\textbf{u}}}_\textrm{I} - \dot{{\textbf{u}}}_h, \quad {\textbf{e}}_h^{{\textbf{W}}} = {\textbf{W}}_\textrm{I} - {\textbf{W}}_h, \quad {\textbf{e}}_h^{\Psi } = \Psi _\textrm{I} - \Psi _h. \end{aligned} \end{aligned}$$

A main assumption of this analysis is the correspondence between the interpolants at time $t=0$ and the discrete initial conditions, such that:

$$\begin{aligned} {\textbf{e}}^{\textbf{u}}_h(0)={\textbf{e}}^{\dot{{\textbf{u}}}}_h(0)={\textbf{e}}^{\textbf{W}}_h(0)={\textbf{0}}, \qquad e^\varphi _h(0)=e^\Psi _h(0)=0. \end{aligned}$$

After introducing the auxiliary variables and the additional problems as in the proof of Theorems 1 and 2, we can subtract them to obtain:

16 $$\begin{aligned} \begin{aligned} {\mathscr {M}}_{u}&(\ddot{{\textbf{u}}}-\ddot{{\textbf{u}}}_h, {\textbf{v}}_h) + {\mathscr {M}}_{\varphi }(\varphi - {\varphi }_h, \psi _h) + {\mathscr {M}}_{\varphi , \tau _1}({\dot{\varphi }}-{\dot{\varphi }}_h, \psi _h) + {\mathscr {A}}_{e,h}({\textbf{u}}-{\textbf{u}}_h, {\textbf{v}}_h) \\&+ {\mathscr {A}}_{e,\delta _1,h}(\dot{{\textbf{u}}}-\dot{{\textbf{u}}}_h, {\textbf{v}}_h) + {\mathscr {A}}_{\text {div},h}({\textbf{u}}-{\textbf{u}}_h, {\textbf{v}}_h) + {\mathscr {A}}_{\text {div},\delta _2,h}(\dot{{\textbf{u}}}-\dot{{\textbf{u}}}_h, {\textbf{v}}_h) \\&+ {\mathscr {A}}_{\varphi ,h}(\Psi -\Psi _h, \psi _h) - {\mathscr {C}}_h({\textbf{v}}_h, \varphi - \varphi _h) +{\mathscr {C}}_h({\textbf{u}}-{\textbf{u}}_h, \psi _h) +{\mathscr {C}}_{\tau _2,h}(\dot{{\textbf{u}}}-\dot{{\textbf{u}}}_h, \psi _h) = 0, \end{aligned} \end{aligned}$$

and

17 $$\begin{aligned} \begin{aligned} {\mathscr {M}}_{u}&(\dot{{\textbf{u}}}-\dot{{\textbf{u}}}_h, {\textbf{v}}_h) + {\mathscr {M}}_{\varphi }(\varphi - {\varphi }_h, \psi _h) + {\mathscr {M}}_{\varphi , \tau _1}({\dot{\varphi }}-{\dot{\varphi }}_h, \psi _h) + {\mathscr {A}}_{e,h}({\textbf{W}}-{\textbf{W}}_h, {\textbf{v}}_h) \\&+ {\mathscr {A}}_{e,\delta _1,h}({{\textbf{u}}}-{{\textbf{u}}}_h, {\textbf{v}}_h) + {\mathscr {A}}_{\text {div},h}({\textbf{W}}-{\textbf{W}}_h, {\textbf{v}}_h) + {\mathscr {A}}_{\text {div},\delta _2,h}({{\textbf{u}}}-{{\textbf{u}}}_h, {\textbf{v}}_h) \\&+ {\mathscr {A}}_{\varphi ,h}(\Psi -\Psi _h, \psi _h) - {\mathscr {C}}_h({\textbf{v}}_h, \Psi - \Psi _h) +{\mathscr {C}}_h({\textbf{u}}-{\textbf{u}}_h, \psi _h) +{\mathscr {C}}_{\tau _2,h}(\dot{{\textbf{u}}}-\dot{{\textbf{u}}}_h, \psi _h) = 0. \end{aligned} \end{aligned}$$

Now, in (16) we take $({\textbf{v}}_h, \psi _h) = (\tau _2 {\textbf{e}}^{\dot{{\textbf{u}}}}_h, e^\varphi _h)$ and multiply everything by $\tau _2$, then we exploit the coercivity and continuity properties of the bilinear forms:

$$\begin{aligned} \begin{aligned} \dfrac{1}{2}\dfrac{\textrm{d}}{\textrm{d}t}&\Big (\Vert \tau _2\sqrt{\rho }{\textbf{e}}^{\dot{{\textbf{u}}}}_h\Vert ^2 + \Vert \tau _2 {\textbf{e}}^{\textbf{u}}_h\Vert _\textrm{dG,e}^2 + \Vert \sqrt{\tau _2 \tau _1 d_0} e^\varphi _h\Vert ^2 + \Vert \sqrt{\tau _2} {e}^\Psi _h\Vert _\mathrm {dG,\varphi }^2\Big ) + \Vert \sqrt{\tau _2 d_0} e^\varphi _h\Vert ^2 \\&+ \Vert \tau _2 {\textbf{e}}^{\dot{{\textbf{u}}}}_h\Vert _\mathrm {dG,\delta }^2 +{\mathscr {C}}_h(\tau _2 {\textbf{e}}^{{{\textbf{u}}}}_h, e^\varphi _h) \le \Vert \tau _2 \sqrt{\rho } {\textbf{e}}^{\ddot{{\textbf{u}}}}_\textrm{I}\Vert \Vert \tau _2 \sqrt{\rho }{\textbf{e}}^{\dot{{\textbf{u}}}}_h\Vert + \Vert \sqrt{\tau _2 d_0} e^\varphi _\textrm{I}\Vert \,\Vert \sqrt{\tau _2 d_0}e^\varphi _h\Vert \\&+ \Vert \sqrt{\tau _2 \tau _1 d_0} e^{{\dot{\varphi }}}_\textrm{I}\Vert \, \Vert \sqrt{\tau _2 \tau _1 d_0} e^\varphi _h\Vert + \dfrac{\textrm{d}}{\textrm{d}t} \Big ({\mathscr {A}}_{e,h}(\tau _2{\textbf{e}}^{{{\textbf{u}}}}_\textrm{I}, \tau _2 {\textbf{e}}^{{{\textbf{u}}}}_h) + {\mathscr {A}}_{\text {div},h}(\tau _2{\textbf{e}}^{{{\textbf{u}}}}_\textrm{I}, \tau _2 {\textbf{e}}^{{{\textbf{u}}}}_h)\Big ) \\  &+|\!\!\,|\!\!\,|\tau _2{\textbf{e}}^{\dot{{\textbf{u}}}}_\textrm{I}|\!\!\,|\!\!\,|_\textrm{dG,e} \Vert \tau _2 {\textbf{e}}^{{{\textbf{u}}}}_h\Vert _\textrm{dG,e} +|\!\!\,|\!\!\,|\tau _2{\textbf{e}}^{\dot{{\textbf{u}}}}_\textrm{I}|\!\!\,|\!\!\,|_\mathrm {dG,\delta } \Vert \tau _2 {\textbf{e}}^{\dot{{\textbf{u}}}}_h\Vert _\mathrm {dG,\delta } + \dfrac{\textrm{d}}{\textrm{d}t} {\mathscr {A}}_{\varphi ,h}(\tau _2e^\Psi _\textrm{I}, e^\Psi _h) \\  &+ \Vert \sqrt{\tau _2}e^\varphi _\textrm{I}|\!\!\,|\!\!\,|_\mathrm {dG,\varphi } \Vert \sqrt{\tau _2}e^\Psi _h\Vert _\mathrm {dG,\varphi } - {\mathscr {C}}_h(\tau _2 {\textbf{e}}^{\dot{{\textbf{u}}}}_h, \tau _2e^\varphi _\textrm{I}) + {\mathscr {C}}_h(\tau _2 {\textbf{e}}^{{{\textbf{u}}}}_\textrm{I}, e^\varphi _h) +{\mathscr {C}}_{\tau _2,h}(\tau _2 {\textbf{e}}^{\dot{{\textbf{u}}}}_\textrm{I}, e^\varphi _h), \end{aligned} \end{aligned}$$

and in (17) we take $({\textbf{v}}_h, \psi _h) =({\textbf{e}}^{\textbf{u}}_h,e^\Psi _h)$:

$$\begin{aligned} \begin{aligned} \dfrac{1}{2}\dfrac{\textrm{d}}{\textrm{d}t}&\Big (\Vert \sqrt{\rho }{\textbf{e}}^{\textbf{u}}_h\Vert ^2 + \Vert \sqrt{d_0} e^\Psi _h \Vert ^2 + \Vert {\textbf{e}}^{\textbf{W}}_h\Vert _\textrm{dG,e}^2 \Big ) + \Vert {\textbf{e}}^{\textbf{u}}_h\Vert _\mathrm {dG,\delta }^2 + \Vert e^\Psi _h\Vert _\mathrm {dG,\varphi }^2 - {\mathscr {C}}_{\tau _2,h}({\textbf{e}}^{{\textbf{u}}}_h, e^\varphi _h) \\&+ \dfrac{\textrm{d}}{\textrm{d}t} {\mathscr {C}}_{\tau _2,h}({\textbf{e}}^{{{\textbf{u}}}}_h, e^\Psi _h) \le \Vert \sqrt{\rho }{\textbf{e}}^{\dot{{\textbf{u}}}}_\textrm{I}\Vert \,\Vert \sqrt{\rho } {\textbf{e}}^{\textbf{u}}_h\Vert + \left( \Vert \sqrt{d_0}{e}^\varphi _\textrm{I}\Vert + \Vert \sqrt{d_0}\tau _1{e}^{{\dot{\varphi }}}_\textrm{I}\Vert \right) \Vert \sqrt{d_0}e^\Psi _h\Vert \\  &- \dfrac{\textrm{d}}{\textrm{d}t}\left( {\mathscr {A}}_{e,h}({\textbf{e}}^{\textbf{W}}_h, {\textbf{e}}^{\textbf{W}}_h)+{\mathscr {A}}_{\textrm{div},h}({\textbf{e}}^{\textbf{W}}_h, {\textbf{e}}^{\textbf{W}}_h) \right) + |\!\!\,|\!\!\,|{\textbf{e}}^{\textbf{u}}_\textrm{I}|\!\!\,|\!\!\,|_\textrm{dG,e} \Vert {\textbf{e}}^{\textbf{W}}_h\Vert _\textrm{dG,e} \\  &+ |\!\!\,|\!\!\,|{\textbf{e}}^{\textbf{u}}_\textrm{I}|\!\!\,|\!\!\,|_\mathrm {dG,\delta } \Vert {\textbf{e}}^{\textbf{u}}_h\Vert _\mathrm {dG,\delta } + |\!\!\,|\!\!\,|e^\Psi _\textrm{I} |\!\!\,|\!\!\,|_\mathrm {dG,\varphi } \Vert e^\Psi _h\Vert _\mathrm {dG,\varphi } - {\mathscr {C}}_h({\textbf{e}}^{\textbf{u}}_h, e^\Psi _\textrm{I}) \\  &+ {\mathscr {C}}_h({\textbf{e}}^{\textbf{u}}_\textrm{I}, e^\Psi _h) - {\mathscr {C}}_{\tau _2,h}({\textbf{e}}^{{\textbf{u}}}_\textrm{I}, e^\varphi _h) + \dfrac{\textrm{d}}{\textrm{d}t} {\mathscr {C}}_{\tau _2,h}({\textbf{e}}^{{{\textbf{u}}}}_\textrm{I}, e^\Psi _h) - {\mathscr {M}}_{\varphi , \tau _1}({e}^{{\dot{\varphi }}}_h, e^\Psi _h). \end{aligned} \end{aligned}$$

Adapting the proof of the stability (see Theorem 1), we will adopt PoincarÃ¨ and Korn’s inequalities to bound some terms in their discrete version (cf. [22, 37]). To handle the last term on the left-hand side, we derive a new estimate from (17) by $(\tau _2{\textbf{e}}^{\textbf{u}}_h,0)$ and we obtain:

$$\begin{aligned} \begin{aligned} \dfrac{1}{2}\dfrac{\textrm{d}}{\textrm{d}t}&\left( \Vert \sqrt{\tau _2\rho } {\textbf{e}}^{\textbf{u}}_h\Vert ^2 + \Vert \sqrt{\tau _2}{\textbf{e}}^{\textbf{W}}_h\Vert _\textrm{dG,e}^2 \right) + \Vert \sqrt{\tau _2}{\textbf{e}}^{\textbf{u}}_h\Vert _\mathrm {dG,\delta }^2 \le {\mathscr {C}}_h(\tau _2{\textbf{e}}^{\textbf{u}}_h, e^\Psi _h) - {\mathscr {C}}_h(\tau _2{\textbf{e}}^{\textbf{u}}_h, e^\Psi _\textrm{I}) \\ +&\Vert \sqrt{\tau _2\rho }{\textbf{e}}^{\dot{{\textbf{u}}}}_\textrm{I}\Vert \;\Vert \sqrt{\tau _2\rho }{\textbf{e}}^{\textbf{u}}_h\Vert + |\!\!\,|\!\!\,|{\textbf{e}}^{\textbf{W}}_\textrm{I}|\!\!\,|\!\!\,|_\textrm{dG,e} \Vert \tau _2{\textbf{e}}^{\textbf{u}}_h\Vert _\textrm{dG,e} + |\!\!\,|\!\!\,|\sqrt{\tau _2}{\textbf{e}}^{\textbf{u}}_\textrm{I}|\!\!\,|\!\!\,|_\mathrm {dG,\delta } \Vert \sqrt{\tau _2}{\textbf{e}}^{\textbf{u}}_h\Vert _\mathrm {dG,\delta }. \end{aligned} \end{aligned}$$

Summing up the two equations and integrating in time, we obtain the following estimate, where we neglect the dependence on the physical parameters (using also the equivalence between the two norms $\Vert \cdot \Vert _{\textrm{dG,e}}$ and $\Vert \cdot \Vert _{\mathrm {dG,\delta }}$):

$$\begin{aligned} \begin{aligned} \dfrac{\textrm{d}}{\textrm{d}t}&\left( \Vert {\textbf{e}}^{\textbf{u}}_h\Vert ^2 + \Vert e^\Psi _h\Vert ^2 + \Vert {\textbf{e}}^{\textbf{W}}_h\Vert _\textrm{dG,e}^2 \right) + \Vert {\textbf{e}}^{\dot{{\textbf{u}}}}_h\Vert ^2 + \Vert {\textbf{e}}^{\textbf{u}}_h\Vert ^2 + \Vert e^\varphi _h\Vert ^2 + \Vert e^\Psi _h \Vert ^2 + \Vert {\textbf{e}}^{\textbf{u}}_h\Vert _\textrm{dG,e}^2 \\ +&\Vert {\textbf{e}}^{\textbf{W}}_h\Vert _\textrm{dG,e}^2 + \Vert e^\Psi _h\Vert _\mathrm {dG,\varphi }^2 + \int _0^t \left( \Vert e^\varphi _h\Vert ^2 + \Vert {\textbf{e}}^{\textbf{u}}_h\Vert _\textrm{dG,e}^2 + \Vert {\textbf{e}}^{\dot{{\textbf{u}}}}_h\Vert _\textrm{dG,e}^2 + \Vert e^\Psi _h\Vert _\mathrm {dG,\varphi }^2 \right) \\ \lesssim&\; \Vert {\textbf{e}}^{\dot{{\textbf{u}}}}_\textrm{I}\Vert ^2 +\Vert e^\Psi _\textrm{I}\Vert ^2 + |\!\!\,|\!\!\,|{\textbf{e}}^{{{\textbf{u}}}}_\textrm{I}|\!\!\,|\!\!\,|_\textrm{dG,e}^2 +|\!\!\,|\!\!\,|{e}^\Psi _\textrm{I}|\!\!\,|\!\!\,|_\mathrm {dG,\varphi }^2 + |\!\!\,|\!\!\,|{\textbf{e}}^{\textbf{W}}_\textrm{I}|\!\!\,|\!\!\,|_\textrm{dG,e}^2 + \Vert {\textbf{e}}^{\textbf{u}}_h\Vert ^2 \\ +&\int _0^t\left( \Vert {\textbf{e}}^{\ddot{{\textbf{u}}}}_\textrm{I}\Vert ^2 + \Vert {\textbf{e}}^{\dot{{\textbf{u}}}}_\textrm{I}\Vert ^2 + |\!\!\,|\!\!\,|{\textbf{e}}^{\dot{{\textbf{u}}}}_\textrm{I}|\!\!\,|\!\!\,|_\textrm{dG,e}^2 + |\!\!\,|\!\!\,|{\textbf{e}}^{\textbf{u}}_\textrm{I}|\!\!\,|\!\!\,|_\textrm{dG,e}^2 + \Vert {\textbf{e}}^{\dot{{\textbf{u}}}}_h\Vert ^2 + \Vert {\textbf{e}}^{{{\textbf{u}}}}_h\Vert _\textrm{dG,e}^2 + \Vert {\textbf{e}}^{\textbf{u}}_h\Vert ^2 + \Vert {\textbf{e}}^{\textbf{W}}_h\Vert _\textrm{dG,e}^2\right) \\ +&\int _0^t\left( \Vert e^\Psi _\textrm{I}\Vert ^2 + \Vert e^\varphi _\textrm{I}\Vert ^2 + \Vert e^{{\dot{\varphi }}}_\textrm{I}\Vert ^2 + \Vert e^\varphi _\textrm{I}|\!\!\,|\!\!\,|_\mathrm {dG,\varphi }^2 +|\!\!\,|\!\!\,|e^\Psi _\textrm{I} |\!\!\,|\!\!\,|_\mathrm {dG,\varphi }^2 +\Vert e^\varphi _h\Vert ^2 + \Vert e^\Psi _h\Vert _\mathrm {dG,\varphi }^2 + \Vert e^\Psi _h\Vert ^2 \right) . \end{aligned} \end{aligned}$$

Finally, by using Grönwall lemma [54], integrating in time and neglecting the errors connected with the additional variables at the left-hand side, we obtain that:

18 $$\begin{aligned} \begin{aligned} \Vert {\textbf{e}}^{\textbf{u}}_h\Vert ^2 +&\int _0^t \left( \Vert {\textbf{e}}^{\dot{{\textbf{u}}}}_h\Vert ^2 + \Vert {\textbf{e}}^{\textbf{u}}_h\Vert ^2 + \Vert {\textbf{e}}^{\textbf{u}}_h\Vert _\textrm{dG,e}^2 \right) \lesssim (1 + t e^t) \int _0^t\Big (\Vert e_h^\varphi \Vert ^2 + \Vert {\textbf{e}}^{\ddot{{\textbf{u}}}}_\textrm{I}\Vert ^2 + \Vert {\textbf{e}}^{\dot{{\textbf{u}}}}_\textrm{I}\Vert ^2 + |\!\!\,|\!\!\,|{\textbf{e}}^{\dot{{\textbf{u}}}}_\textrm{I}|\!\!\,|\!\!\,|_\textrm{dG,e}^2 \\ +&|\!\!\,|\!\!\,|{\textbf{e}}^{\textbf{u}}_\textrm{I}|\!\!\,|\!\!\,|_\textrm{dG,e}^2 + |\!\!\,|\!\!\,|{\textbf{e}}^{\textbf{W}}_\textrm{I}|\!\!\,|\!\!\,|_\textrm{dG,e}^2 + \Vert e^\Psi _\textrm{I}\Vert ^2 + \Vert e^\varphi _\textrm{I}\Vert ^2 + \Vert e^{{\dot{\varphi }}}_\textrm{I}\Vert ^2 + \Vert e^\varphi _\textrm{I}|\!\!\,|\!\!\,|_\mathrm {dG,\varphi }^2 + |\!\!\,|\!\!\,|e^\Psi _\textrm{I} |\!\!\,|\!\!\,|_\mathrm {dG,\varphi }^2 \Big ). \end{aligned} \nonumber \\ \end{aligned}$$

The thesis follows by bounding the right-hand side of Equation (18) using the interpolation estimates of Proposition 1.
